# Tumor-Infiltrating PD-1^hi^CD8^+^-T-Cell Signature as an Effective Biomarker for Immune Checkpoint Inhibitor Therapy Response Across Multiple Cancers

**DOI:** 10.3389/fonc.2021.695006

**Published:** 2021-09-15

**Authors:** Zhenyu Yang, Yulan Deng, Jiahan Cheng, Shiyou Wei, Hao Luo, Lunxu Liu

**Affiliations:** ^1^Institute of Thoracic Oncology and Department of Thoracic Surgery, West China Hospital, Sichuan University, Chengdu, China; ^2^Western China Collaborative Innovation Center for Early Diagnosis and Multidisciplinary Therapy of Lung Cancer, Sichuan University, Chengdu, China

**Keywords:** immune checkpoint inhibitor, cancer, PD-1^hi^CD8^+^ T cell, biomarker, CXCL13

## Abstract

**Background:**

Stratification of patients who could benefit from immune checkpoint inhibitor (ICI) therapy is of much importance. PD-1^hi^CD8^+^ T cells represent a newly identified and effective biomarker for ICI therapy response biomarker in lung cancer. Accurately quantifying these T cells using commonly available RNA sequencing (RNA-seq) data may extend their applications to more cancer types.

**Method:**

We built a transcriptome signature of PD-1^hi^CD8^+^ T cells from bulk RNA-seq and single-cell RNA-seq (scRNA-seq) data of tumor-infiltrating immune cells. The signature was validated by flow cytometry and in independent datasets. The clinical applications of the signature were explored in non-small-cell lung cancer, melanoma, gastric cancer, urothelial cancer, and a mouse model of breast cancer samples treated with ICI, and systematically evaluated across 21 cancer types in The Cancer Genome Atlas (TCGA). Its associations with other biomarkers were also determined.

**Results:**

Signature scores could be used to identify the PD-1^hi^CD8^+^ T subset and were correlated with the fraction of PD-1^hi^CD8^+^ T cells in tumor tissue (Pearson correlation, R=0.76, *p*=0.0004). Furthermore, in the scRNA-seq dataset, we confirmed the capability of PD-1^hi^CD8^+^ T cells to secrete CXCL13, as well as their interactions with other immune cells. In 581 clinical samples and 204 mouse models treated with ICIs, high signature scores were associated with increased survival, and the signature achieved area under the receiver operating characteristic curve scores of 0.755 (ranging from 0.61 to 0.91) in predicting therapy response. In TCGA pan-cancer datasets, our signature scores were consistently correlated with therapy response (R=0.78, *p*<0.0001) and partially explained the diverse response rates among different cancer types. Finally, our signature generally outperformed other mRNA-based predictors and showed improved predictive performance when used in combination with tumor mutational burden (TMB). The signature score is available in the R package “PD1highCD8Tscore” (https://github.com/Liulab/PD1highCD8Tscore).

**Conclusion:**

Through estimating the fraction of the PD-1^hi^CD8^+^ T cell, our signature could predict response to ICI therapy across multiple cancers and could serve as a complementary biomarker to TMB.

## Introduction

Genomic alterations in malignant tumors distinguish them from normal cells and produce persistent antigenic stimulation, thereby suppressing T cell functions (1–3). Immune checkpoint inhibitors (ICIs) successfully reinvigorate T cell functions and have led to impressive progress in the treatment of non-small-cell lung cancer (NSCLC), melanoma, and urothelial cancer, especially in the advanced stages (4–6). However, only a limited proportion of patients receiving ICI therapy have superior clinical outcomes across various cancer types. To solve this problem, several biomarkers have been identified in recent years, including tumor mutational burden (TMB), tumor-neoantigen burden, programmed cell death protein 1 (PD-1) or programmed cell death ligand 1 (PD-L1) expression level, interferon-gamma (IFNγ) signature, and CD8^+^ T cell infiltration (7–11). Although these factors are related to the effectiveness of ICIs, their predictive power is not sufficient (9, 12), nor do they fully explain the mechanism of resistance to ICIs. There is thus an urgent need for new biomarkers that can be used to identify patients sensitive to ICI therapy

PD-1^hi^CD8^+^ T cells represent a distinct population of CD8^+^ T cells, which are upregulated in T-cell-exhaustion and cell proliferation process (13). A recent retrospective analysis used immunohistochemistry (IHC) assays to estimate the fraction of PD-1^hi^CD8^+^ T cells in the tumor microenvironment (TME) and demonstrated that this was positively associated with treatment response and patient survival in cases of NSCLC treated with PD-1 blockade (13). This finding raised the question of whether the predictive value of PD-1^hi^CD8^+^ T cells could be extrapolated to other cancer types. Commonly available datasets such as RNA sequencing (RNA-seq) datasets could help to settle this issue. Therefore, we built a transcriptional signature for PD-1^hi^CD8^+^ T cells, validated its ability to quantify such cells in the TME, and further explored its predictive performance with respect to ICI therapy outcomes across multiple cancer types.

## Materials and Methods

### Data Collection

We identified ICI-treated patients with available RNA-seq data from the Gene Expression Omnibus (GEO, https://www.ncbi.nlm.nih.gov/geo/) and Sequence Read Archive (SRA, https://www.ncbi.nlm.nih.gov/sra/) databases. Data from The Cancer Genome Atlas (TCGA, http://cancergenome.nih.gov/;legacy archive, version 2016_01_28) were downloaded from FIREBROWSE (http://firebrowse.org/). All the deposited datasets are summarized in **Table 1**.

**Table 1:** Key resource table.

Resource	Source	Identifier
**Deposited Data**		
Solid tumor samples	CCLE	https://portals.broadinstitute.org/
Immune cell from healthy individuals	DICE	https://dice-database.org/
Pan-cancer tumor samples (21 types)	TCGA	http://firebrowse.org/
Sorted PD-1^hi/low/neg^CD8^+^ T cells from NSCLC	Thommen et al. (13)	SRA: SRP108393
Sorted PD-1^hi/low/neg^CD8^+^ T cells from HCC, flow cytometry, and RNA-seq results	Kim et al. (14)	GEO: GSE111389
Sorted PD-1^hi/low/neg^CD8^+^ T cells from breast cancer	Guo et al. (15)	SRA: SRP189910
Anti-PD-1/anti-PD-1 combined with anti-CTLA4 treated melanoma (Gide)	Gide et al. (16)	ENA: ERP105482
Anti-PD-1 treated melanoma (Riaz)	Riaz et al. (17)	SRA: SRP094781
Anti-PD-1 treated gastric cancer	Kim et al. (18)	ENA: ERP107734
Anti-PD-L1 treated urothelial cancer	Mariathasan et al. (5)	http://research-pub.gene.com/IMvigor210CoreBiologies/
Anti-PD-1/PD-L1 treated NSCLC (Jung)	Jung et al. (19)	GEO: GSE135222
Anti-PD-1 treated NSCLC (Cho)	Cho et al. (20)	GEO: GSE126044
ScRNA-seq of immune cells in melanoma TME	Sade-Feldman et al. (21)	GEO: GSE120575
ScRNA-seq of T cells in NSCLC TME	Guo et al. (22)	GEO: GSE99254
Anti-PD1/anti-CTLA4 combination therapy treated mouse model of breast cancer	Hollern et al. (23)	GEO: GSE124821
Human genome (GRCH38/hg38)	Genome Reference Consortium	http://www.ncbi.nlm.nih.gov/projects/genome/assembly/grc/human
**Software and Algorithms**		
STAR version 2.5.4b	Dobin et al. (24)	https://github.com/alexdobin/STAR
Stringtie, version v1.3.4d	Pertea et al. (25)	https://ccb.jhu.edu/software/stringtie/
Metascape	Zhou et al. (26)	https://metascape.org/
CellPhoneDB	Efremova et al. (27)	https://github.com/Teichlab/cellphonedb
MSigDB	–	http://www.gsea-msigdb.org/gsea/msigdb
CIBERSORT	Newman et al. (28)	https://cibersort.stanford.edu/
quanTIseq	Finotello et al. (29)	https://github.com/icbi-lab/quanTIseq
**R packages/scripts**		
Codes used for scoring	This paper	https://github.com/Liulab/PD1highCD8Tscore
DESeq2 (1.26.0)	Love et al. (30)	https://bioconductor.org/packages/release/bioc/html/DESeq2.html
limma (3.41.16)	Ritchie et al. (31)	https://bioconductor.org/packages/release/bioc/html/limma.html
singscore (1.10.0)	Foroutan et al. (32)	https://www.bioconductor.org/packages/release/bioc/html/singscore.html
Seurat (3.1.0)	Butler et al. (33)	https://satijalab.org/seurat/
edgeR (3.27.13)	Robinson et al. (34)	https://bioconductor.org/packages/release/bioc/html/edgeR.html
fgsea (1.12.0)	Sergushichev (35)	http://bioconductor.org/packages/release/bioc/html/fgsea.html
pROC (1.15.3)	Robin et al. (36)	https://cran.r-project.org/web/packages/pROC/index.html
survival (3.1-12)	–	https://cran.r-project.org/web/packages/survival/index.html
survminer (0.4.8)	–	https://cran.r-project.org/web/packages/survminer/index.html
biomaRt (2.42.0)	Durinck et al. (37)	https://bioconductor.org/packages/release/bioc/html/biomaRt.html
immunophenoscore	Charoentong et al. (38)	https://tcia.at/tools/toolsMain

### Identification of Differentially Expressed Genes

The RNA-seq data were aligned to the human genome (GRCH38/hg38) using the STAR (24) version 2.5.4b 2-pass mapping strategy. Transcript assembly and gene level quantification were performed using StringTie version v1.3.4d (25). DEGs were identified using DESeq2 (30) (version 1.26.0). Genes were considered to be DEGs based on adjusted p value (*p*.adj) < 0.05 and |log2 [fold change (FC)] | >1.

### Construction of Gene Signature for PD-1^hi^CD8^+^ T Cells

**Figure 1:**
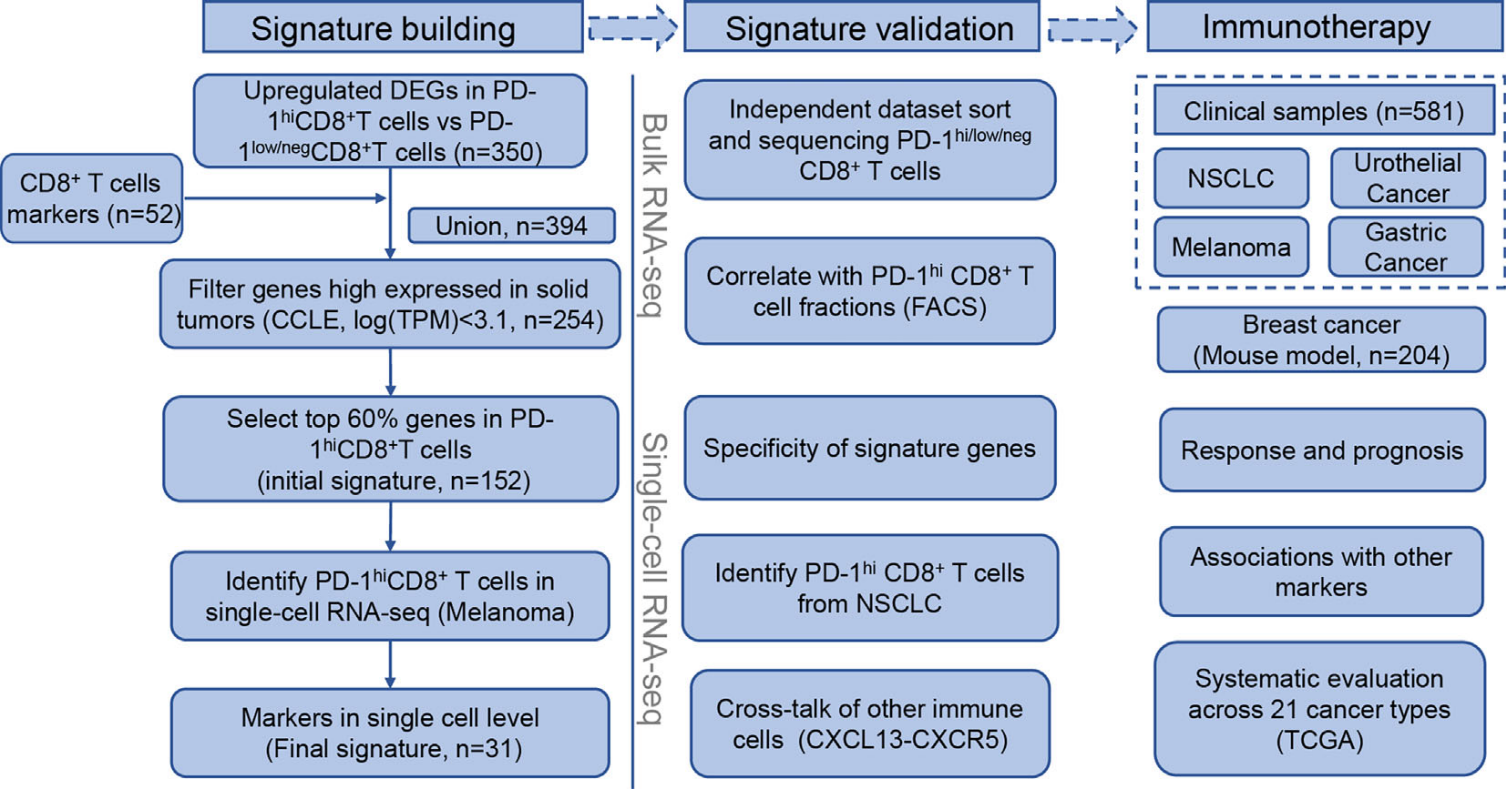
Flowchart of PD-1^hi^CD8^+^ T cells signature building, validation, and clinical implications in immune checkpoint inhibitor therapy.

The workflow followed to build the signature building is described in **Figure 1** (left). First, we combined CD8^+^-T-cell-specific genes from Schmiedel et al. (39) with PD-1^hi^CD8^+^-T-cell-specific genes from NSCLC tumor-infiltrating lymphocytes (TILs) (13). In the latter case, PD-1^hi^CD8^+^-T-cell-specific genes were defined as genes that were significantly upregulated in PD-1^hi^CD8^+^ T cells compared with other CD8^+^ T cells. Second, we excluded genes that were highly expressed in solid tumors using expression data from The Cancer Cell Line Encyclopedia (CCLE, https://portals.broadinstitute.org/ccle). Genes were retained if their median expression (log transcripts per million [TPM]) in cancer cells was below 3.1. Third, to determine the optimal threshold for filtering low expression genes in PD-1^hi^CD8^+^ T cells, we scanned a range of cutoffs to select top genes as input for unsupervised hierarchical clustering (from 20% to 80%, with 20% increments). We found that the top 60% genes were sufficient to distinguish PD-1^hi^CD8^+^ T cells from other CD8^+^ T cells and kept them as the initial gene set. The signature scores were calculated by singscore (32), where the background gene set was selected as genes with mean TPM>1 in TCGA samples (21 cancer types). When calculating the signature score of the initial gene set in a single-cell RNA-seq(scRNA-seq) dataset (GSE120575) (21), we found that a cluster of CD8^+^ T cells resembled PD-1^hi^CD8^+^ T cells from NSCLC TILs (13). Therefore, we kept the cluster marker genes from the initial signature genes and got a reduced final signature. The discrimination abilities of the initial and final gene signature scores were compared using the area under the receiver operation characteristic curve (AUC). Finally, we supplied an easy-to-use R package “PD1highCD8Tscore” to calculate our signature score.

### Signature Validation

To explore whether the signature genes were specific to PD-1^hi^CD8^+^ T cells, we compared the expression levels of signature genes between PD-1^hi^CD8^+^ T cells and other immune cells in an external dataset (39) after normalization by 15 house-keeping genes (RPL38, UBA52, RPL4, RPS29, SLC25A3, CLTC, RPL37, PSMA1, RPL8, PPP2CA, TXNL1, MMADHC, PSMC1, RPL13A, and MRFAP1) (40). Two previous studies also sorted and sequenced PD-1^hi/low^/^neg^CD8^+^ T cells among hepatocellular carcinoma (HCC) and breast cancer TILs with a similar gating and sorting strategy (14, 15). To estimate whether our signature characterized similar cell populations in NSCLC, breast cancer, and HCC, we performed principal component analysis (PCA) and calculated the correlations between all cells after removing the batch effect using the “limma” package (31). Scores were calculated and compared in each bulk RNA-seq dataset. Moreover, we calculated the scores in 17 HCC tumor samples and analyzed the correlation between scores and the absolute fraction of PD-1^hi^CD8^+^ T cells. The absolute fraction of PD-1^hi^ CD8^+^ T cells was defined as the product of the relative fraction of PD-1^hi^ CD8^+^ T cells in CD8^+^ T cells and the fraction of CD8^+^ T cells in tumor samples. The former fraction was based on the flow cytometry results of Kim et al. (data were obtained by personal communications). The latter fraction was estimated by QuanTIseq (29).

We downloaded TPM data and/or count data and cell labels from two scRNA-seq (21, 22) datasets for immune cells. Cells with 1,000–5,000 detected genes and expressing <5% mitochondrial genes were retained. Standard procedures for variable gene selection, dimensionality reduction, and clustering were performed using Seurat version 3 (33), and the top 3,000 variable genes were selected. Signature scores were calculated based on TPM data. The cluster with the highest signature score was labeled the PD-1^hi^CD8^+^ T cell, and other clusters were labeled according to the original paper. Clustering results were visualized using uniform manifold approximation and projection. Differential expression test was performed using the “FindMarkers” function with Wilcoxon rank-sum test in genes expressed in at least 25% of cells using the default threshold (|logFC|>0.25). Gene ontology (GO) enrichment analyses were performed using Metascape (26) with *p*<0.01 and enrichment score >1.5. Cell–cell communication analysis was conducted using CellPhoneDB (27).

### Signature Scores in ICI-Treated Patients and Mouse Models

We scored all ICI-treated samples (5, 16–20) and compared the differences between responders (durable clinical benefit, DCB) and nonresponders (nondurable benefit, NDB) by Wilcoxon rank-sum test. DCB was defined as complete response (CR), partial response (PR), or stable disease (SD) for more than 6 months. NDB was defined as progressive disease (PD) or SD for less than 6 months. In two studies (17, 18), no detailed information was available on DCB/NDB; therefore, we compared the CR/PR group with the PD group as a surrogate. In a mouse model of ICI-treated breast cancer (23), we used the “biomaRt” package (37) to convert genes from mouse to human and compared the scores between ICI-resistant and ICI-sensitive samples. The predictive value of the signature score in response for ICI therapy was evaluated by calculating AUC values (36). Patients with survival data available were divided into high and low score groups according to the Yuden index. Overall survival (OS) and progression-free survival (PFS) were estimated by Kaplan-Meier curves, and the log-rank test was used to compare Kaplan-Meier survival curves. A multivariable Cox proportional hazard model was built to correct the effects of potential confounding factors.

### TCGA Data Processing

Reported objective response rates (ORRs) across 21 cancer types were obtained from Lee and Ruppin (9). We scored 6,764 samples from TCGA in the corresponding cancer types. We investigated the correlation of the proportion of high signature score samples in each cancer type with the response rate. DEGs between the samples with the top 33% and bottom 33% signature scores were detected by edgeR (34). The definition of DEGs was the same as that in Section 2.2. Kyoto Encyclopedia of Genes and Genomes (KEGG) gene sets from the Molecular Signatures Database (MSigDB, http://www.gsea-msigdb.org/gsea/msigdb) were scored for DEGs using gene set variation analysis (GSVA), where *p*<0.05 and |normalized enrichment score (NES)| >1 were considered to indicate significance.

### Other ICI Therapy Biomarkers

We compared our signature with other predictive biomarkers for ICI. PD-L1 is an IHC biomarker approved by the Food and Drug Administration (FDA) (41). PD-L1 gene expression was used here as an IHC surrogate. PD-1 gene expression is also a predictor of ICI therapy response (7). IFNγ was found to be a response biomarker by Ayers et al. (10). The mean expression of six genes (IFNG, STAT1, IDO1, CXCL10, CXCL9, and HLA-DRA) in this pathway was used to estimate their performance (10). The score for Anti-CTLA4 resistance MAGE genes (CRMA) was calculated as the average of MAGEA3, MAGEA2, MAGEA2B, MAGEA12, and MAGEA6 (42). Immunophenoscore (IPS) was calculated using a script supplied by The Cancer Immunome Atlas (https://tcia.at/) (38). Tertiary lymphoid structure (TLS) signature genes were obtained from Cabrita et al. (43). CD8^+^ T cell proportions were estimated by CIBERSORT (28) (CD8^+^ T CIBERSROT). TMB was available for two cohorts. For **Figures 6A, B**, a logistic regression of TMB and signature score was used to assess the combined predictive value for ICI. In the survival analysis of **Figures 6C–F**, patients were divided into four groups, TMB^high^Score^high^, TMB^low^Score^high^, TMB^high^Score^low^, and TMB^low^Score^low^, according to their TMB and signature score. The cutoff was determined by the best Yuden index. Similarly, the effect of combination of PDL1 and our score was also analyzed.

### Statistical Analysis

All the software packages and algorithms are summarized in **Table 1**. R version 3.5.1 was used for statistical analysis and visualization. The AUC was used to evaluate the predictive value of the signature score to ICI therapy. A two-sided Wilcoxon rank-sum test was used for between-group comparisons, and *p*-value<0.05 was regarded as statistically significant.

## Results

### Building the PD-1^hi^CD8^+^-T-Cell-Derived Signature

The flowchart of the process of building the PD-1^hi^CD8^+^-T-cell signature is depicted in **Figure 1** (left). We first combined CD8^+^-T-cells-specific genes and DEGs in PD-1^hi^CD8^+^ T cells (**Supplementary Figure 1**), resulting in 394 genes. To identify immune-specific genes, the sequencing data of solid cancer from CCLE were downloaded, and 150 genes highly expressed (logTPM>3.1) in cancer cells were filtered. We selected the top 60% expressed genes in PD-1^hi^CD8^+^ T cells as an initial signature (152 genes), which were sufficient for clustering PD-1^hi^CD8^+^ T cells apart from PD-1^low/neg^CD8^+^ T (**Supplementary Figures 2**, **3A, B**). In a scRNA-seq dataset, we found a cluster of cells with a high initial signature score that exhibited a similar phenotype to that of PD-1^hi^CD8^+^ T cells (upregulated genes enriched in T cell exhaustion and cell proliferation/growth) (**Supplementary Figures 4A, B**). The common genes between the initial signature and marker genes of this cluster were considered as the final signature (31 genes, **Table S1**). The discrimination ability of the final signature was the same as that of the initial signature (AUC=1) (**Supplementary Figures 3A, B**). The signature included seven cell-cycle-associated genes (BARD1, CENPE, RAD51, SMC2, GINS2, CLSPN, and CCNF) and seven T-cell-exhaustion-associated genes (CTLA4, PDCD1, TOX, SIRPG, HAVCR2, TIGIT, and IGFLR1) (44, 45).

### Validation of PD-1^hi^CD8^+^-T-Cell-Derived Signature

**Figure 2:**
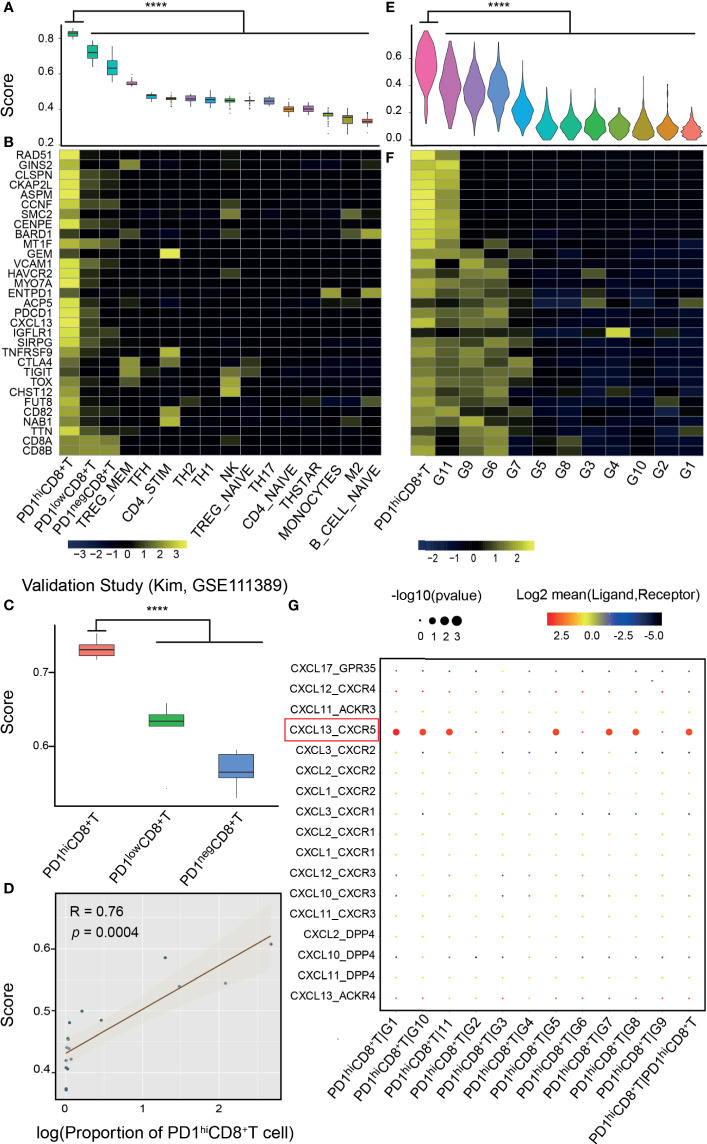
Validation of the signature score from bulk and single-cell RNA-seq data. **(A)** Bulk RNA-seq data of PD-1^hi^CD8^+^ T cells and other immune cells from the database of immune cell expression project were integrated after normalization by house-keeping genes. PD-1^hi^CD8^+^ T cells had the highest score than other immune cells (****: *p*<0.0001). **(B)** Signature genes were specific to PD-1^hi^CD8^+^ T cells. **(C)** The signature score can discriminate PD-1^hi/low/neg^CD8^+^ T cells in another study (18), where these cells were sorted and sequenced from hepatocellular carcinoma (HCC). **(D)** The proportion of PD-1^hi^CD8^+^ T cells in HCC tumor samples was correlated with the signature score (Pearson correlation, R=0.76, *p*=0.0004). **(E)** In single-cell RNA-seq of melanoma tumor infiltrating immune cells, PD-1^hi^CD8^+^ T cells had the highest score (****: *p*<0.0001), and the signature genes were highly expressed in this cluster **(F)**. **(G)** The ligand–receptor interactions between PD1^hi^CD8^+^T cells and other immune cells. PD1^hi^CD8^+^T cells secreted CXCL13 chemokine and interacted with the chemokine receptor CXCR5 expressing on B cells, cytotoxicity lymphocytes, memory T cells, and regulatory T cells. G1, B cells; G2, plasma cells; G3, monocytes/macrophages; G4, dendritic cells; G5, lymphocytes; G6, exhausted CD8^+^ T cells; G7, regulatory T cells; G8, cytotoxicity lymphocytes; G9, exhausted/heat-shock CD8^+^ T cells; G10, memory T cells; G11, exhausted/cell cycle (CD4^+^ T cell).

The PD-1^hi^CD8^+^-T-cell signature was validated in both bulk RNA-seq and scRNA-seq data (**Figure 1**, middle). In the bulk RNA-seq data (13, 39), we found that the signature score could discriminate PD-1^hi^CD8^+^ T cells from other immune cells collected from healthy individuals (*p*<0.0001), and the signature genes were relatively specific (**Figures 2A, B**). In two independent studies (14, 15), PD-1^hi/low/neg^CD8^+^ T cells were also sorted and sequenced from HCC and breast cancer TILs by flow cytometry. The PCA and correlation results showed that the sorted PD-1^hi^CD8^+^ T cells had similar transcriptional features and were tissue-agnostic among lung, liver, and breast tissues (**Supplementary Figures 3C, D**). The PD-1^hi^CD8^+^ T cells had the highest signature score compared with other CD8^+^ T cells and were accurately identified by our score (AUC=1) (**Figure 2C** and **Supplementary Figures 3A, B, E**). The signature score was correlated with the fraction of PD-1^hi^CD8^+^ T cells in HCC tumor samples (Pearson correlation, R=0.76, *p*=0.0004, **Figure 2D**). Patients with high fractions of PD-1^hi^CD8^+^ T cells had higher scores than other patients (*p*=0.0009, **Supplementary Figure 3F**). In scRNA-seq data of melanoma TILs (21), the PD-1^hi^CD8^+^ T cells were identified by our score (**Figure 2E** and **Supplementary Figure 4A**), and most of the signature genes had higher expression values in this subset than other immune cells in the TME. (**Figure 2F**). Similar results were found in another scRNA-seq data of NSCLC TILs (**Supplementary Figure 4C**) (22). We identified a high-scoring subcluster of exhausted CD8^+^ T cells, with highly expressed genes enriched in cell proliferation/growth (**Supplementary Figure 4B**). GSVA analyses also confirmed that the cell cycle pathway was more activated in PD-1^hi^CD8^+^ T cells compared with other exhausted CD8^+^ T cell subsets with PD-1 expression (melanoma: NES=2.07, *p*.adj=0.0059; NSCLC: NES=1.98, *p*.adj=0.0130). Increased glycolysis (melanoma: *p*<0.0001; NSCLC: *p*<0.0001) and secretion of CXCL13 were found in (melanoma: logFC=1.22, *p*.adj<0.0001; NSCLC: logFC=0.85, *p*.adj<0.0001) PD-1^hi^CD8^+^ T cell, consistent with a previous report (**Supplementary Figure 5**) (13). The cell–cell interaction analysis using CellPhoneDB found that PD-1^hi^CD8^+^ T cells interacted with B cells (mean= 5.424, *p*<0.0001), regulatory T cell (mean= 3.801, *p*<0.0001), and cytotoxicity T cells (mean= 3.683, *p*<0.0001) through the CXCL13-CXCR5 axis (**Figure 2G**).

### Effectiveness of Signature Score in Predicting Response to ICI Therapy

**Figure 3:**
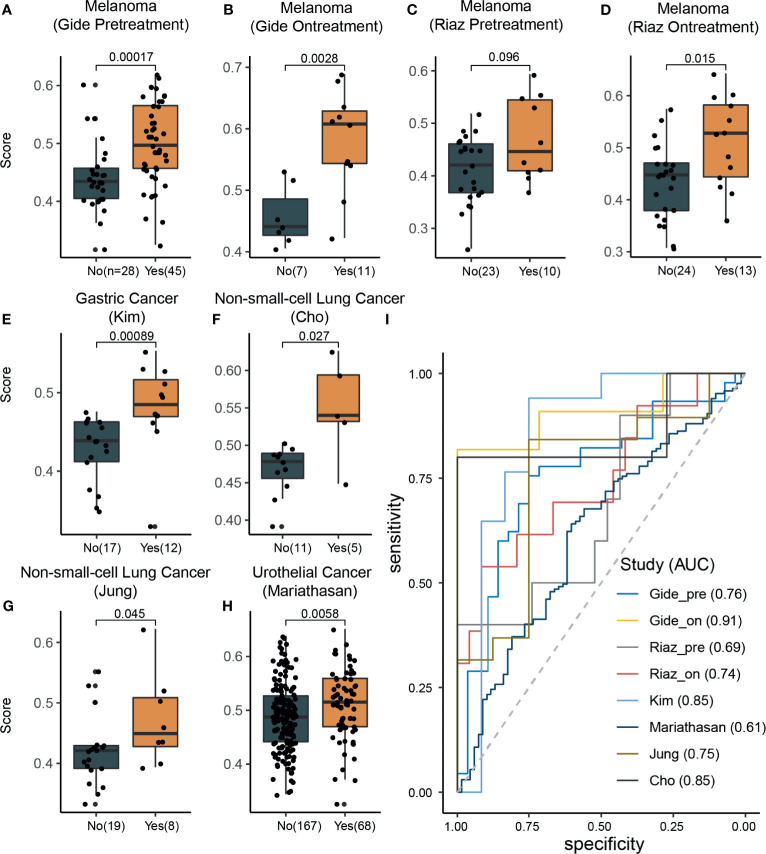
The signature scores were associated with response rates across different cancer types. **(A–H)** In all datasets, the response group samples had significant (Wilcoxon rank-sum test) higher signature scores than nonresponse group samples (marginally significant in the Riaz pretreatment dataset; **C**). **(I)** Receiver operating characteristic curve analysis for response prediction in all datasets.

**Figure 4:**
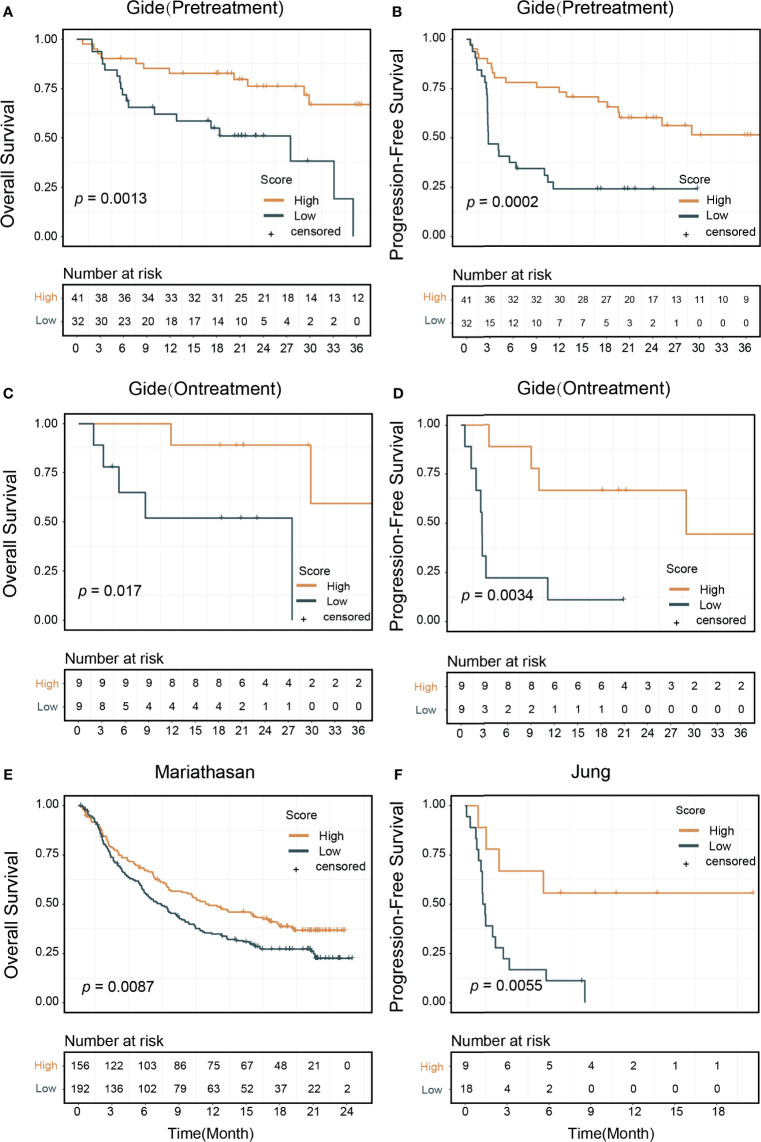
Signature score and patients survival outcomes. Patients treated with immune checkpoint inhibitors were divided into high and low signature score groups. Kaplan–Meier curves showed that high score group patients had better survival outcomes: **(A)** In the pretreatment Gide dataset, *p*=0.013 (log-rank test) in overall survival, **(B)** *p*=0.0002 in progression-free survival. **(C)** In the on-treatment Gide dataset, *p*=0.017 for overall survival, **(D)** *p*=0.0034 in progression-free survival. **(E)** In the Mariathasan dataset, *p*=0.0087 for overall survival. **(F)** In the Jung dataset, *p*=0.0055 for progression-free survival.

To evaluate the effectiveness of our signature score in predicting response to ICI therapy, we obtained 581 RNA-seq samples from eight cohorts (six studies) across four cancer types, NSCLC, melanoma, gastric cancer, and urothelial cancer (**Figure 1**, right). These studies are summarized in **Supplementary Table 2**. Interestingly, the signature scores were significantly higher in responders than in nonresponders, except in one cohort where the difference was nearly significant (*p*=0.096) (**Figures 3A–H**). In another 204 ICI-treated breast cancer mouse models, the sensitive groups had higher scores than resistant groups at all the 4 timepoints of sample collection (pretreatment, *p*<0.0001;3 days after ICI *p*=0.0002;7 days after ICI *p*=0.0001; end of study *p*<0.0001; **Supplementary Figure 6**). Next, we calculated AUCs to evaluate the predictive value of PD-1^hi^CD8^+^ T cells for ICI therapy response. The median AUC across the eight cohorts was 0.755 (range: 0.61 to 0.91) (**Figure 3I**). In two studies involving both pretreatment and on-treatment datasets, the AUCs in the on-treatment groups were higher than those in the pretreatment group from the same study. Moreover, the signature score (continuous variable, **Table S3**) was positively associated with OS and PFS. We divided each cohort into high- and low-score groups and found that high-score patients had better survival with respect to either OS or PFS (**Figure 4**). Furthermore, to explore whether the signature score was an independent prognostic factor, we built a multivariable Cox proportional hazard model including age, sex, and treatment regimens if available. A high PD-1^hi^CD8^+^ T cell score was found to be independently associated with a 76–94% reduction in the risk of disease progression and a 28–92% reduction in the risk for all mortality (**Table 2**). Similarly, the hazard ratios (HRs) in the on-treatment cohort (OS, HR=0.08, *p*=0.034; PFS, HR=0.06, *p*=0.003) were smaller than those in the pretreatment group (OS, HR=0.29, *p*=0.002; PFS, HR=0.29, *p*<0.001). Taken together, these results showed that our signature score could predict ICI therapy response and survival outcomes.

**Table 2:** Multivariable Cox proportional model of signature score and other clinical factors.

Gide et al. pretreatment	HR	2.50%CI	97.50%CI	P
	Progression-free survival (n = 73)			
Score (n, %)				
Low (32, 43.8%)	Ref			
High (41, 56.5%)	0.29	0.15	0.56	<0.001
Age (mean ± sd: 61.6 ± 13.8)	0.99	0.97	1.02	0.559
Gender (n, %)				
Female (26, 35.6%)	Ref			
Male (47, 64.4)	1.44	0.74	2.8	0.286
Regimen (n, %)				
Monotherapy (41, 56.2%)	Ref			
Combined-therapy (32, 44.8)	0.43	0.22	0.85	0.015
Gide et al. on-treatment	Progression-free survival (n=18)			
Score (n, %)				
Low (32, 43.8%)				
High (41, 56.5%)	0.06	0.01	0.38	0.003
Age (mean ± sd: 60.3 ± 15.1)	0.97	0.92	1.02	0.295
Gender (n, %)				
Female (5, 27.8%)	Ref			
Male (13, 71.2%)	0.14	0.02	0.87	0.035
Regimen (n, %)				
Monotherapy (9, 50.0%)	Ref			
Combined-therapy (9, 50.0%)	1.38	0.37	5.17	0.63
Gide et al. pretreatment	Overall survival (n=73)			
Score (n, %)				
Low (32, 43.8%)				
High (41, 56.5%)	0.29	0.13	0.64	0.002
Age (mean ± sd: 61.6 ± 13.8)	1.00	0.97	1.03	0.938
Gender(n, %)				
Female (26, 35.6%)	Ref			
Male (47, 64.4)	1.60	0.67	3.83	0.294
Regimen(n, %)				
Monotherapy (41, 56.2%)	Ref			
Combined-therapy (32, 44.8)	0.25	0.09	0.7	0.008
Gide et al. on-treatment	Overall survival (n=18)			
Score(n, %)				
Low (32, 43.8%)	Ref			
High (41, 56.5%)	0.08	0.01	0.83	0.034
Age (mean ± sd: 60.3 ± 15.1)	1.00	0.97	1.03	0.938
Gender(n, %)				
Female (5, 27.8%)	Ref			
Male (13, 71.2%)	1.60	0.67	3.83	0.294
Regimen(n, %)				
Monotherapy (9, 50.0%)	Ref			
Combined-therapy (9, 50.0%)	0.25	0.09	0.70	0.008
Mariathasan et al.	Overall survival (n=348)			
Score (n, %)				
Low (192, 55.2%)	Ref			
High (156, 44.8%)	0.72	0.56	0.94	0.016
Gender (n, %)				
Female (76, 21.8%)	Ref			
Male (272, 78.2%)	0.81	0.60	1.10	0.183
Baseline ECOG (mean ± sd: 0.67 ± 0.57)	1.96	1.53	2.51	0
Smoking History (n, %)				
Current (35, 10.1%)	Ref			
Never (116, 33.3%)	1.18	0.75	1.87	0.472
Previous (197, 56.1%)	1.13	0.73	1.75	0.589
Received Platinum (n, %)				
No (76, 21.8%)	Ref			
Yes (272, 78.2%)	1.80	1.26	2.58	0.001
Jung et al.	Progression-free survival (n=27)			
Score (n, %)				
Low (18, 66.7%)				
High (9, 33.3%)	0.23	0.08	0.71	0.011
Age (mean ± sd: 62.1 ± 9.0)	0.80	0.18	3.52	0.765
Gender (n, %)				
Female (5, 18.5%)	Ref			
Male (22, 81.5%)	0.94	0.31	2.87	0.92

### Predictive Value of the Signature Score in Pan-Cancer Analysis

**Figure 5:**
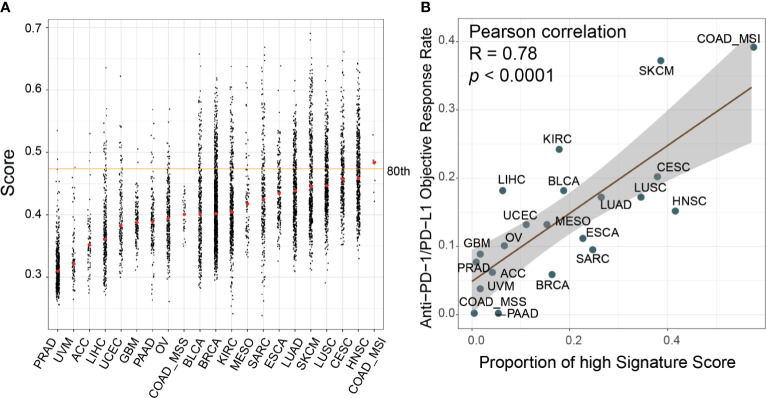
Evaluation of the correlates of immune-check point inhibitors to signature scores across cancer types. **(A)** Distribution of the signature scores across 21 cancer types in The Cancer Genome Atlas (TCGA) dataset. The red dot within each cancer type denoted the median score; the orange line represented the 80th percentile score across all samples. **(B)** The proportion of high signature score (>80^th^ percentile) samples was correlated with the reported objective response rates in a published paper (Pearson correlation, R=0.78, *p*<0.0001). ACC, adrenocortical carcinoma; BLCA, bladder urothelial carcinoma; BRCA, breast invasive carcinoma; CESC, cervical squamous cell carcinoma and endocervical adenocarcinoma; COAD, colon adenocarcinoma; ESCA, esophageal carcinoma; GBM, glioblastoma multiforme; HNSC, head and neck squamous cell carcinoma; KIRC, kidney renal clear cell carcinoma; LIHC, liver hepatocellular carcinoma; LUAD, lung adenocarcinoma; LUSC, lung squamous cell carcinoma; MESO, mesothelioma; OV, ovarian serous cystadenocarcinoma; PAAD, pancreatic adenocarcinoma; PRAD, prostate adenocarcinoma; SARC, sarcoma; SKCM, skin cutaneous melanoma; UCEC, uterine corpus endometrial carcinoma; UVM, uveal melanoma.

We calculated the signature scores of 6,764 samples across 21 cancer types from TCGA and collected ORR data for anti-PD-1/PD-L1 therapy from Lee and Ruppin (9). The distribution of signature scores was higher in immune-hot tumors, including colon adenocarcinoma with microsatellite instability phenotype (COAD_MSI), lung squamous cell carcinoma (LUSC), lung adenocarcinoma (LUAD), head and neck squamous cell carcinoma, skin cutaneous melanoma (SKCM), and bladder urothelial carcinoma (BLCA) which were more sensitive to ICI therapy (**Figure 5**) (46, 47). In prostate adenocarcinoma, ovarian serous cystadenocarcinoma (OV) and glioblastoma multiforme tumors, which are known to be immune-cold tumors, the signature scores were lower (46, 47). Moreover, in the Mariathasan dataset, the immune-inflamed phenotype had higher scores than the immune-desert and immune-excluded phenotypes (**Supplementary Figure 7A**). Generally, the breast cancer samples showed low immune infiltration but had scores at the median level. This phenomenon could be attributed to the heterogeneity of tumor subtypes; triple-negative breast cancer had higher scores than other subtypes (*p*<0.0001, **Supplementary Figure 7B**). The fraction of high-score (80th-percentile) samples was correlated with ORR (Pearson correlation, R=0.78, *p*<0.0001). To explore the differences between high-score (top 33%) and low-score (bottom 33%) samples, DEGs were identified and their KEGG pathway enrichment was analyzed (**Supplementary Figure 8**). T cell receptor signaling pathway, cytokine–cytokine receptor interaction, and chemokine signaling pathway were activated in samples with high signature score across most cancer types (85%, [17/20], 100% [20/20], and 85%[17/20], respectively). The cell adhesion molecule (CAM) pathway, an important mediator of immune cell migration (48), was also upregulated in high-score samples (85%[17/20]). These results demonstrated that cancers with high signature scores had increased cell cross-talk and immune cell infiltration, reflecting an immune-hot phenotype. We also found that glycolysis and fatty acid metabolism were upregulated only in some ICI-therapy-sensitive cancer types. The interaction of PD-1 with the PD-L1 axis can upregulate aerobic glycolysis and induce fatty acid oxidation in T cells, leading to immunosuppression (49). Consequently, these cancer types were sensitive to anti-PD-1/PD-L1 therapy.

### Comparison With Other ICI Biomarkers

**Figure 6:**
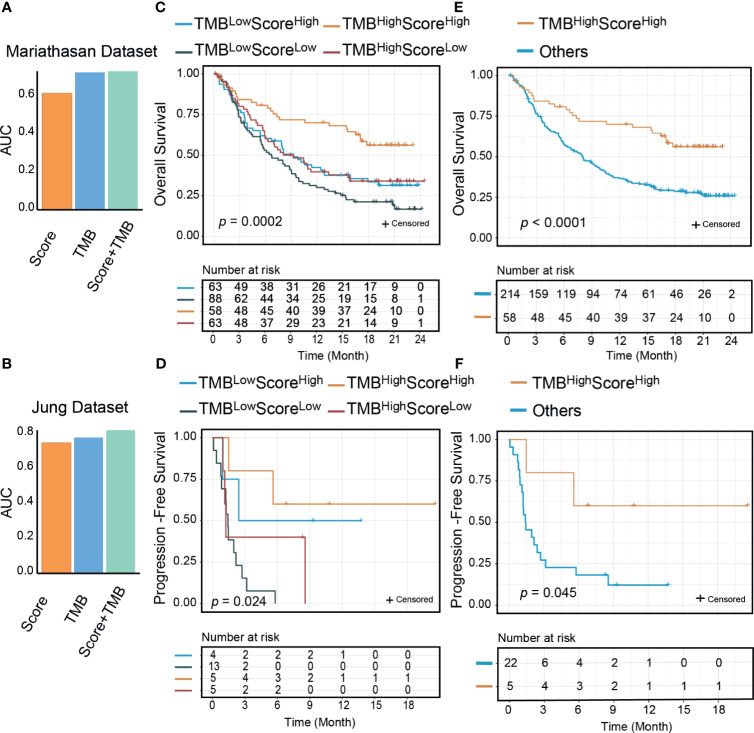
The signature score could improve the predictive and prognostic value of tumor mutational burden (TMB). The area under the receiver operating characteristic curve (AUC) of the combination of the signature score and TMB was higher than TMB and the signature score (**A**: Mariathasan dataset; **B**: Jung dataset); Significant differences exist between the four groups (TMB^high^Score^high^, TMB^low^Score^high^, TMB^high^Score^low^, and TMB^low^Score^low^) in either overall survivals (**C**: Mariathasan dataset, *p*=0.0002) or progression-free survivals (**D**: Jung dataset, *p*=0.024) than other groups. TMB^high^Score^high^ group patients had better overall survival (**E**: Mariathasan dataset, *p*<0.0001) and progression-free survival (**F**: Jung dataset, *p*=0.045) than other three groups.

We compared the predictive performance of our signature score with those of other widely used transcriptome biomarkers, including IFN-γ, PD-L1, PD-1, CRMA, TLS, IPS, and CD8^+^ T CIBERSORT. Our score was positively associated with all of these markers except CRMA (**Supplementary Figure 9A**). The net reclassification index showed that our score could better classify responders to ICI therapy except in two Riaz cohorts (**Supplementary Figure 9B**). A possible explanation was that in the Riaz cohorts, some of the patients had received ipilimumab therapy before PD-1 treatment, which may have influenced their immune status. The AUCs of the signature score, IFN-γ, and PD-L1 were higher than those of the other biomarkers (**Supplementary Figure 9C**). PD-L1 mRNA expression was correlated with our score (**Supplementary Figures 9A**, **10A–H**). In many cases (n=452), PD-L1 and our score stratified patients consistently (**Supplementary Table 4**). In the Mariathasan cohort, where there were 75 discordant samples between PD-L1 and our signature score, the combination of PD-L1 and our score resulted in slightly improved predictive value (AUC from 0.61 to 0.63). It is noteworthy that the OS rate was significantly higher for PD-L1^high^Score^high^ samples than for other samples (log-rank, *p*=0.0025) (**Supplementary Figures 10I–K**). TMB has been reported as a genomic predictor of ICI therapy response in multiple cancer types (9). In two cohorts where TMB data were available, we found no significant association between TMB and our score (**Supplementary Figure 9A**). The combination of our signature and TMB was of higher predictive value than either our signature or TMB alone (**Figures 6A, B**). Next, we divided the patients into four groups by our signature score and TMB. The 1-year OS rates were 70.0% (95% CI: 59.0-83.0%) for the TMB^high^Score^high^ group, 39.7% (95% CI: 29.0–54.3%) for the TMB^high^Score^low^ group, 42.4% (95% CI: 31.8–56.7%) for the TMB^low^Score^high^ group, and 30.1% (95% CI: 21.7–41.7%) for the TMB^low^Score^low^ group (Mariathasan cohort, **Figure 6C**). The 1-year PFS rates were 60% (95% CI: 29–100%) for the TMB^high^Score^high^ group and 50.0% (95% CI: 18.8–100%) for the TMB^low^Score^high^, in the other two groups; all patients progressed in less than 1 year (Jung cohort, **Figure 6D**). Patients in the TMB^high^Score^high^ group showed better survival outcomes compared with other groups (log-rank p<0.001 for OS, p=0.045 for PFS, **Figures 6E, F**). In the Mariathasan cohort, patients in the TMB^high^Score^high^ group showed increased OS compared with patients in the TMB^high^Score^low^ group (log-rank *p*=0.008; HR=0.51, 95%CI: 0.30-0.84, *p*=0.008), suggesting that the signature score could act as a complementary biomarker to TMB.

## Discussion

The introduction of ICI therapy represents a milestone in cancer therapy. However, the low response rates to this type of therapy will increase the economic burden on cancer patients. Treatment with anti-PD-1 agents requires about $6,000 per infusion, which is a much higher cost than transcriptome tests. Therefore, developing biomarkers for predicting ICI therapy response is an urgent and cost-effective field. In this study, we built a transcriptional signature of PD-1^hi^CD8^+^ T cells. The signature score could discriminate this population from other immune cells in both bulk and single-cell-level datasets across different cancer types. Based on flow cytometry and RNA-seq results from an independent study, we validated the ability of our signature to quantify the fraction of PD-1^hi^CD8^+^ T cells in tumor samples. Furthermore, we found that in NSCLC, melanoma, gastric cancer, urothelial cancer, and a mouse model of breast cancer, samples with high signature scores showed more benefit from ICI therapy. The predictive value of the signature score was better than those of other transcriptional markers. Combination of the signature score with TMB would improve its predictive value. In 21 TCGA cancer types, the signature score was correlated with ICI therapy response, revealing the intrinsic connection of the immunological activity of the TME described by the signature of each cancer tissue. Our study also offers an easy-to-use R package to evaluate PD-1^hi^CD8^+^ T cell infiltration in more tumor samples.

Many studies have revealed that CD8^+^ TILs are heterogenous, and that different types of CD8^+^ TILs showed different response to ICIs (1, 21, 44). Our results also demonstrated the limited predictive value of CD8^+^ TILs. Persistent antigen exposure from tumor cells or antigen-presenting cells can cause CD8^+^ T cell exhaustion, inducing sustained expression of PD-1 in CD8^+^ TILs (1). Even within this dysfunctional compartment, CD8^+^ T cells have distinct roles in tumor immunity. Thommen et al. found that in NSCLC, the presence of PD-1^hi^CD8^+^ T cells strongly predicted response to ICI therapy and was correlated with increased OS (13). In these cells, immune-checkpoint genes (PD-1, CTLA4, TIGIT, HAVCR2/TIM-3, and TNFRSF9) (8) and transcription factor TOX, a central regulator of distinct exhausted T cell transformation (45), were highly expressed. PD-1^hi^CD8^+^ T cells were distinct from other PD-1 positive CD8^+^ T cells, as the cell cycle and glycolysis process were upregulated in these cells. This phenotype was similar to that of the PD-1^+^TIM-3^+^ T_RM_ cell identified by Clarke et al. (50), which was shown to be a favorable ICI therapy marker in NSCLC (50). Although it was not clear to what extent PD-1^hi^CD8^+^ T cells overlapped with PD-1^+^TIM-3^+^ T_RM_ cells, the predictive values of PD-1^hi^CD8^+^ T cells were confirmed across 5 cancer types in nearly 600 clinical samples and 204 mouse models. Generally, we found that PD-1^hi^CD8^+^ T cell scores of on-treatment samples were better than those of pretreatment samples because of the better reflection of immune status after therapy (51). Two studies have reported that the relative fraction of PD-1^hi^CD8^+^ T cells in CD8^+^ cells, rather than the absolute fraction in total tumor tissue as estimated by our signature score, was negatively associated with ICI therapy outcomes (21, 52). In predicting ICI therapy response, the relative fraction of PD-1^hi^CD8^+^ T cells might be largely influenced by the complex functions of other intratumor CD8^+^ T cells with negative or low PD-1 expression (21, 53).

Our results indicated that the PD-1^hi^CD8^+^ T cell score was associated with the immune phenotype. Immune-hot tumors (COAD_MSI, SKCM, BLCA, LUSC, and LUAD) had higher signature scores and were sensitive to ICI therapy. Our score was predictive of the ORR to anti-PD-1/PD-L1 therapy across 21 cancer types in 6,764 TCGA samples (Pearson correlation, R=0.78, *p*<0.0001). In support of these results, we found that important pathways in antitumor activity were consistently upregulated in high-score samples, including cytokine–cytokine receptor interaction, chemokine signaling pathway, and T cell receptor signaling pathway (54). The CAM pathway was also activated in the high-score group, which was correlated with T cell infiltration in tumors (48). Moreover, in the Mariathasan dataset, immune-inflamed tumors had higher signature scores than immune-excluded or immune-desert samples. However, not all scores for these tumors were correlated with their phenotype. This discordance could be related to the spatial location of PD-1^hi^CD8^+^ T cells and the impact of other immune cells, whereas our study mainly focused on the transcriptional features of PD-1^hi^CD8^+^ T cells. Heterogeneity of tumor subtypes could also weaken the associations between our score and immune phenotypes. For example, breast cancer is generally considered as low immune-reactive cancer, but triple-negative breast cancer has been reported to be a high-immune-infiltration subtype (55). Our results consistently showed that this subtype had higher scores than other subtypes. Similarly, the COAD_MSI subtype was of immune-hot phenotype, unlike other COAD subtypes (56). On the other hand, glycolysis and fatty acid metabolism were only upregulated in some of the ICI-sensitive cancer samples with high scores. In PD-1 signaling, tumor cells can block antitumor immunity through this two metabolic pathways (49). These results partially explain why these cancer types are sensitive to anti-PD-1/PD-L1 therapy. Recent findings showed that targeting these metabolic interventions in combination with ICI could offer opportunities to improve therapy response; several clinical trials of such treatment are ongoing (57).

The main function of PD-1^hi^CD8^+^ T cells is the secretion of CXCL13. In a previous study, CXCL13 was found to be mainly expressed by CD4^+^ follicular helper T cells, recruiting B cells and inducing TLS formation in the nonlymphoid tissue (58). In scRNA-seq data, as well as identifying a cluster of CD4^+^ T cells with high CXCL13 expression, we confirmed the secretion of CXCL13 from PD-1^hi^CD8^+^ T cells and their interaction with B cells and regulatory T cells through the CXCL13-CXCR5 axis. These results indicated that PD-1^hi^CD8^+^ T cells might recruit and organize immune cells through secretion of CXCL13, modulating the TME to an immune-hot phenotype. The positive association between the TLS score and the PD-1^hi^CD8^+^ T cell score also supports this. The importance of CXCL13 in ICI therapy has been confirmed *in vivo*. Anti-PD-1 therapy failed in a CXCL13-null mouse model of BLCA, whereas the wild-type model showed a good response (59), and the treatment with a combination of CXCL13 and anti-PD-1 successfully retarded tumor growth in another mouse model of OV (60). Although the differences in CXCL13 secretion between CD4^+^ T cells and PD-1^hi^CD8^+^ T cells remain elusive, these results indicate that CXCL13 is a potential therapeutic target that could be targeted in combination with ICI therapy.

Increasing evidence shows that response to ICI therapy is influenced by both immune cells and tumor-associated factors. PD-L1 positivity of tumor cells has been shown to be a good indicator of response to ICI therapy. In recent years, it has been recognized that high PD-L1 expression in dendritic cells, regulatory T cells, and macrophages can attenuate T cell activation and promote T cell exhaustion (61). The expression of PD-1 or PD-L1 in the TME is important for ICI therapy. In our study, patients in the PD-L1^high^Score^high^ group benefited more from ICI therapy than other patients (**Supplementary Figure 10K**). TMB-high tumors had high levels of neoantigens, which make them more immunogenic and trigger a TIL response (11, 12). TMB has been approved by the FDA as a genomic biomarker in some cancer types (41, 62). However, TMB has some limitations as a biomarker, and it has been suggested that a combination of TMB with other predictors may show superior performance (12). Whereas TMB is reflective of tumor properties, our signature score for the PD-1^hi^CD8^+^ T cell characterizes the tumor environment. In our analysis, TMB and the signature score both showed a good predictive value for ICI but were independent of each other. Therefore, it was intuitive to explore their potential combined effects. When samples were divided into four subtypes based on TMB and the signature score, the patients in the TMB^high^Score^high^ group not only were highly immunogenic (characterized by a high TMB) but also showed an immune hot phenotype (characterized by a high signature score). These patients would be expected to be more sensitive to ICI therapy, and indeed they exhibited the best clinical outcomes in two independent datasets. These results warrant further confirmation and extension.

One of the potential limitations of our study was that our analysis was an integrated, retrospective study. Second, although the multivariable Cox proportional hazard model showed that the PD-1^hi^CD8^+^ T cell score was an independent prognostic indicator, some clinical prognostic factors, including the number of metastatic sites, first-line therapy information, and details of ICI therapy regimens, were not available. In the Riaz cohort, the difference between responders and nonresponders was not as obvious as those in other cohorts. Some patients receiving ipilimumab therapy before PD-1 treatment might be an additional confounder that would weaken the statistical power of our analysis (63, 64). Third, our study did not explore the mechanisms underlying the different effects of the PD-1^hi^CD8^+^ T cell score in different cohorts or the variation in cutoff values for high-score samples in different cohorts. One possible mechanism may involve the complicated PD-L1 status in tumor cells or immune cells, like dendritic cells and macrophages. However, the gene expression of PD-L1 is a mixture of those cell types in RNA-seq; other methods including cytometry by time of flight and codetection by indexing may help explore this in the future.

## Conclusions

In summary, we built a 31-gene signature to represent the fraction of PD-1^hi^CD8^+^ T cells from bulk RNA-seq data and demonstrated promising potential of the PD-1^hi^CD8^+^ T cell as a pan-cancer biomarker in patients receiving ICI therapy. The combination of the signature score with TMB could further increase its predictive value. The secretion of CXCL13 is a potential mechanism of how PD-1^hi^CD8^+^ T cells modulate the TME and why high-scoring patients tend to have favorable outcomes of ICI therapy.

## Data Availability Statement

The original contributions presented in the study are included in the article/**Supplementary Material**. Further inquiries can be directed to the corresponding author.

## Author Contributions

Study concept and design: ZY, YD, and LL. Acquisition, analysis, or interpretation of data: ZY, YD, JC, SW, and HL. Drafting of the manuscript: all authors. Critical revision of the manuscript for important intellectual content: all authors. Study supervision: LL and YD. All authors contributed to the article and approved the submitted version.

## Funding

This study was funded by the National Natural Science Foundation of China (NSFC 81672311 to LL and NSFC 31801119 to YD).

## Conflict of Interest

The authors declare that the research was conducted in the absence of any commercial or financial relationships that could be construed as a potential conflict of interest.

## Publisher’s Note

All claims expressed in this article are solely those of the authors and do not necessarily represent those of their affiliated organizations, or those of the publisher, the editors and the reviewers. Any product that may be evaluated in this article, or claim that may be made by its manufacturer, is not guaranteed or endorsed by the publisher.

## Abbreviations

AUC, area under the receiver operation characteristic curve; BLCA, bladder urothelial carcinoma; CAM, cell adhesion molecules; CCLE, The Cancer Cell Line Encyclopedia; CR, complete response; CRMA, Anti-CTLA4 resistance MAGE genes; COAD_MSI, colon adenocarcinoma with microsatellite instability phenotype; DCB, durable clinical benefit; DEG, differential expressed gene; FDA, Food and Drug Administration; GO, gene ontology; GSVA, gene set variation analysis; HCC, hepatocellular carcinoma; ICI, immune checkpoint inhibitor; IHC, immunohistochemistry; IPS, immunophenoscore; KEGG, Kyoto Encyclopedia of Genes and Genomes; LUAD, lung adenocarcinoma; LUSC, lung squamous cell carcinoma; MSigDB, Molecular Signatures Database; NSCLC, non-small cell lung cancer; NDB, nondurable benefit; ORR, objective response rate; OS, overall survival; OV, ovarian serous cystadenocarcinoma; PFS, progression-free survival; PR, partial response; scRNA-seq, single-cell RNA-seq; SD, stable disease; SKCM, skin cutaneous melanoma; TCGA, The Cancer Genome Atlas; TIL, tumor infiltrating lymphocytes; TMB, tumor mutational burden.
