# Supplementary material for: Tumor-Infiltrating PD-1hiCD8+-T-Cell Signature as an Effective Biomarker for Immune Checkpoint Inhibitor Therapy Response Across Multiple Cancers
Source: Front Oncol. 2021 Sep 15;11:695006. doi: 10.3389/fonc.2021.695006 (PMC8479164; doi:10.3389/fonc.2021.695006)
Supplement: Supplementary file 1 [file DataSheet_1.docx]

**Supplementary Materials**

**Supplementary Tables**

**Supplementary Table 1. PD1^hi^CD8^+^T-cell-signature contains 31 genes**

| Gene Name | Gene ID | Description | Annotation |
| --- | --- | --- | --- |
| RAD51 | 5888 | RAD51 recombinase | Cell Cycle |
| GINS2 | 51659 | GINS complex subunit 2 | Cell Cycle |
| CLSPN | 63967 | claspin | Cell Cycle |
| CKAP2L | 150468 | cytoskeleton associated protein 2 like |  |
| ASPM | 259266 | assembly factor for spindle microtubules |  |
| CCNF | 899 | cyclin F | Cell Cycle |
| SMC2 | 10592 | structural maintenance of chromosomes 2 | Cell Cycle |
| CENPE | 1062 | centromere protein E | Cell Cycle |
| BARD1 | 580 | BRCA1 associated RING domain 1 | Cell Cycle |
| MT1F | 4494 | metallothionein 1F |  |
| GEM | 2669 | GTP binding protein overexpressed in skeletal muscle |  |
| VCAM1 | 7412 | vascular cell adhesion molecule 1 |  |
| HAVCR2 | 84868 | hepatitis A virus cellular receptor 2 | T cell exhaustion |
| MYO7A | 4647 | myosin VIIA |  |
| ENTPD1 | 953 | ectonucleoside triphosphate diphosphohydrolase 1 |  |
| ACP5 | 54 | acid phosphatase 5, tartrate resistant |  |
| PDCD1 | 5133 | programmed cell death 1 | T cell exhaustion |
| CXCL13 | 10563 | C-X-C motif chemokine ligand 13 | Recruiting immune cells |
| IGFLR1 | 79713 | IGF like family receptor 1 | T cell exhaustion |
| SIRPG | 55423 | signal regulatory protein gamma | T cell exhaustion |
| TNFRSF9 | 3604 | TNF receptor superfamily member 9 |  |
| CTLA4 | 1493 | cytotoxic T-lymphocyte associated protein 4 | T cell exhaustion |
| TIGIT | 201633 | T cell immunoreceptor with Ig and ITIM domains | T cell exhaustion |
| TOX | 9760 | thymocyte selection associated high mobility group box | T cell exhaustion |
| CHST12 | 55501 | carbohydrate sulfotransferase 12 |  |
| FUT8 | 2530 | fucosyltransferase 8 |  |
| CD82 | 3732 | CD82 molecule |  |
| NAB1 | 4664 | NGFI-A binding protein 1 |  |
| TTN | 7273 | titin |  |
| CD8A | 925 | CD8a molecule |  |
| CD8B | 926 | CD8b molecule |  |

**Supplementary Table 2 . Summary of included ICI therapy studies**

| **Cohort** | **Study** | **Cancer** | **Time** | **No.** | **Regimen** | **Outcomes** | **Clinical variables** | **Ref.** |
| --- | --- | --- | --- | --- | --- | --- | --- | --- |
| 1 | Gide | Melanoma | Pretreatment | 73 | Anti-PD-1/Anti-PD-1 combined with anti-CTLA4 | Response, OS, PFS | Age, sex | 24 |
| 2 |  |  | On-treatment | 18 |  |  |  |  |
| 3 | Riaz | Melanoma | Pretreatment | 33* | Anti-PD-1 | Response | - | 25 |
| 4 |  |  | On-treatment | 37* |  |  |  |  |
| 5 | Kim | Gastric cancer | Pretreatment | 29* | Anti-PD-1 | Response | - | 26 |
| 6 | Mariathasan | Urothelial Cancer | Pretreatment | 348 | Anti-PD-L1 | Response, OS | Age, sex, smoking history, immune phenotype, race, ECOG, platinum, TMB, metastasis site. | 4 |
| 7 | Jung | NSCLC | Pretreatment | 27 | Anti-PD-1/PD-L1 | Response, PFS | Age, sex, TMB | 27 |
| 8 | Cho | NSCLC | Pretreatment | 16 | Anti-PD-1 | Response | - | 28 |

**Abbreviations:** ECOG, Eastern Cooperative Oncology Group performance status; TMB, tumor mutational burden. NSCLC, non-small-cell lung cancer.

*** :** Patients in stable disease without lasting time were excluded.

**Supplementary Table 3 Univariable Cox proportional analysis of signature score (continuous variable) in ICI therapy**

| Cohort | HR | 2.50% CI | 97.50% CI | P |
| --- | --- | --- | --- | --- |
| Gide et al. pretreatment (PFS, n=18) | 0.58 | 0.42 | 0.79 | <0.001 |
| Gide et al. ontreatment (PFS, n=18) | 0.37 | 0.18 | 0.76 | 0.007 |
| Gide et al. pretreatment (OS, n=73) | 0.54 | 0.37 | 0.80 | 0.002 |
| Gide et al. ontreatment (OS, n=18) | 0.28 | 0.09 | 0.89 | 0.030 |
| Mariathasan et al. (OS, n=348) | 0.86 | 0.75 | 0.97 | 0.02 |
| Jung et al. (PFS, n=27) | 0.61 | 0.35 | 1.06 | 0.05 |

**Supplementary Table 4. The associations between PD-L1 and our score in stratifying patients.**

|  |  | **Score** | |  |  |  | **Score** | |  |
| --- | --- | --- | --- | --- | --- | --- | --- | --- | --- |
| **Cohort** | **PDL1** | **High** | **Low** | **P** | **Cohort** | **PDL1** | **High** | **Low** | **P** |
| **1** | **High** | 30 | 5 | <0.001 | **5** | **High** | 9 | 9 | 0.004 |
|  | **Low** | 11 | 27 |  |  | **Low** | 1 | 10 |  |
| **2** | **High** | 9 | 0 | <0.001 | **6** | **High** | 117 | 36 | <0.001 |
|  | **Low** | 0 | 9 |  |  | **Low** | 39 | 156 |  |
| **3** | **High** | 4 | 9 | 0.017 | **7** | **High** | 6 | 1 | 0.017 |
|  | **Low** | 0 | 20 |  |  | **Low** | 3 | 17 |  |
| **4** | **High** | 9 | 13 | 0.005 | **8** | **High** | 3 | 1 | 0.027 |
|  | **Low** | 0 | 15 |  |  | **Low** | 1 | 11 |  |
| **All** | **High** | 187 | 74 |  |  |  |  |  |  |
|  |  | 55 | 265 |  |  |  |  |  |  |

**Supplementary Figures and Figure Legends**

**
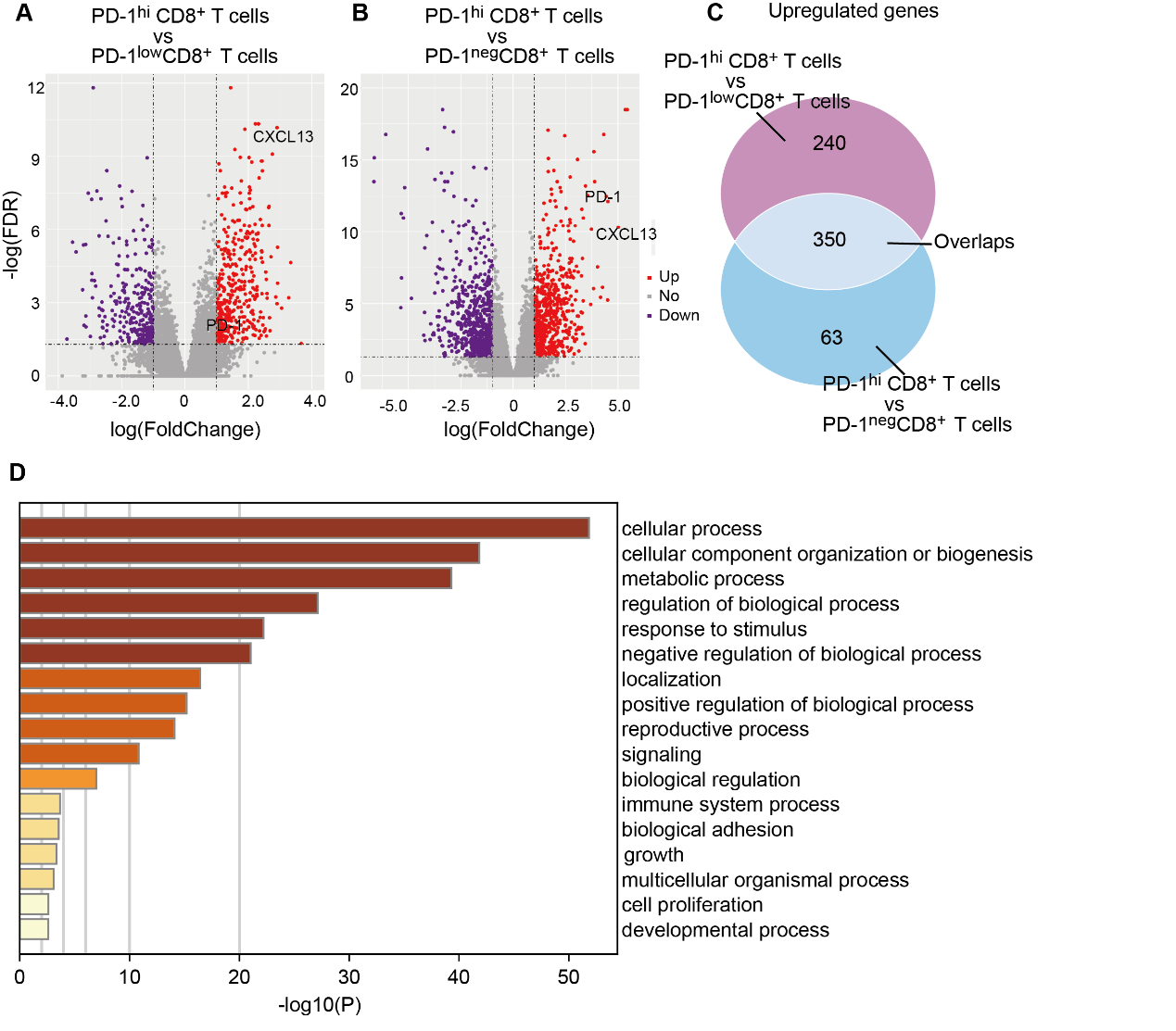
**

**Supplementary Figure 1.** Differential expressed genes (DEGs) analysis of PD-1^hi^CD8^+^ T cells versus PD-1^low/neg^CD8^+^ T cells from Thommen et al. dataset. Volcano plot of DEGs in PD-1^hi^CD8^+^ T cells versus PD-1^low^CD8^+^ T cells(A) and PD-1^hi^CD8^+^ T cells versus PD-1^neg^CD8^+^ T cells(B). (C)The Venn plot showed 350 genes were upregulated in PD-1^hi^CD8^+^ T cells compared to PD-1^low/neg^CD8^+^ T cells. (D) Gene oncology enrichment analysis of 350 upregulated genes in Metascape.


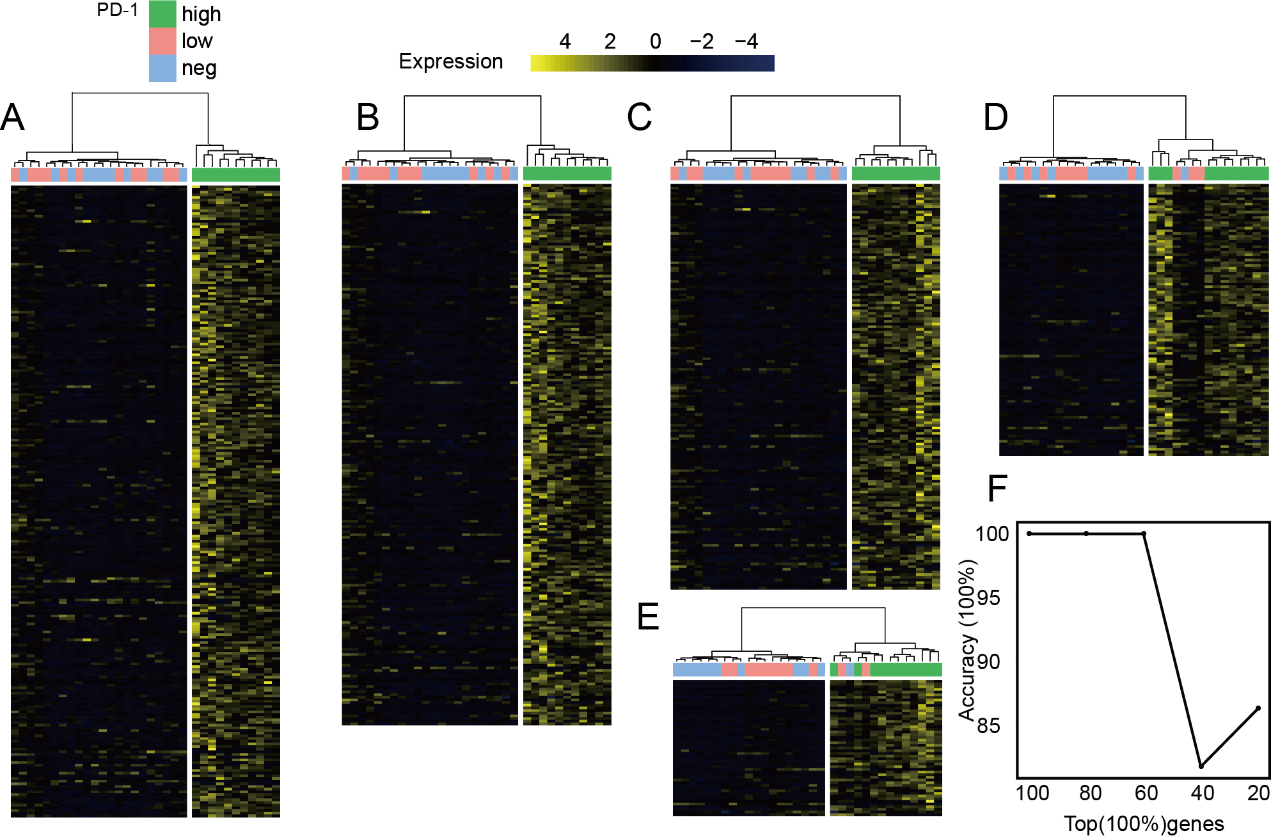


**Supplementary Figure 2.** Appropriate threshold for filtering lowly expressed genes in PD-1^hi^CD8^+^ T cells. The top 100%(A), 80%(B), 60%(C), 40%(D), 20%(E) genes were selected to cluster PD-1^hi/low/neg^CD8^+^ T cells using unsupervised hierarchical clustering, trees were cut using k=2. (F). Accuracy in clustering results. Selecting top 60% genes can distinguish PD-1^hi^CD8^+^ T from PD-1^low/neg^CD8^+^ T cells with minimum gene counts.


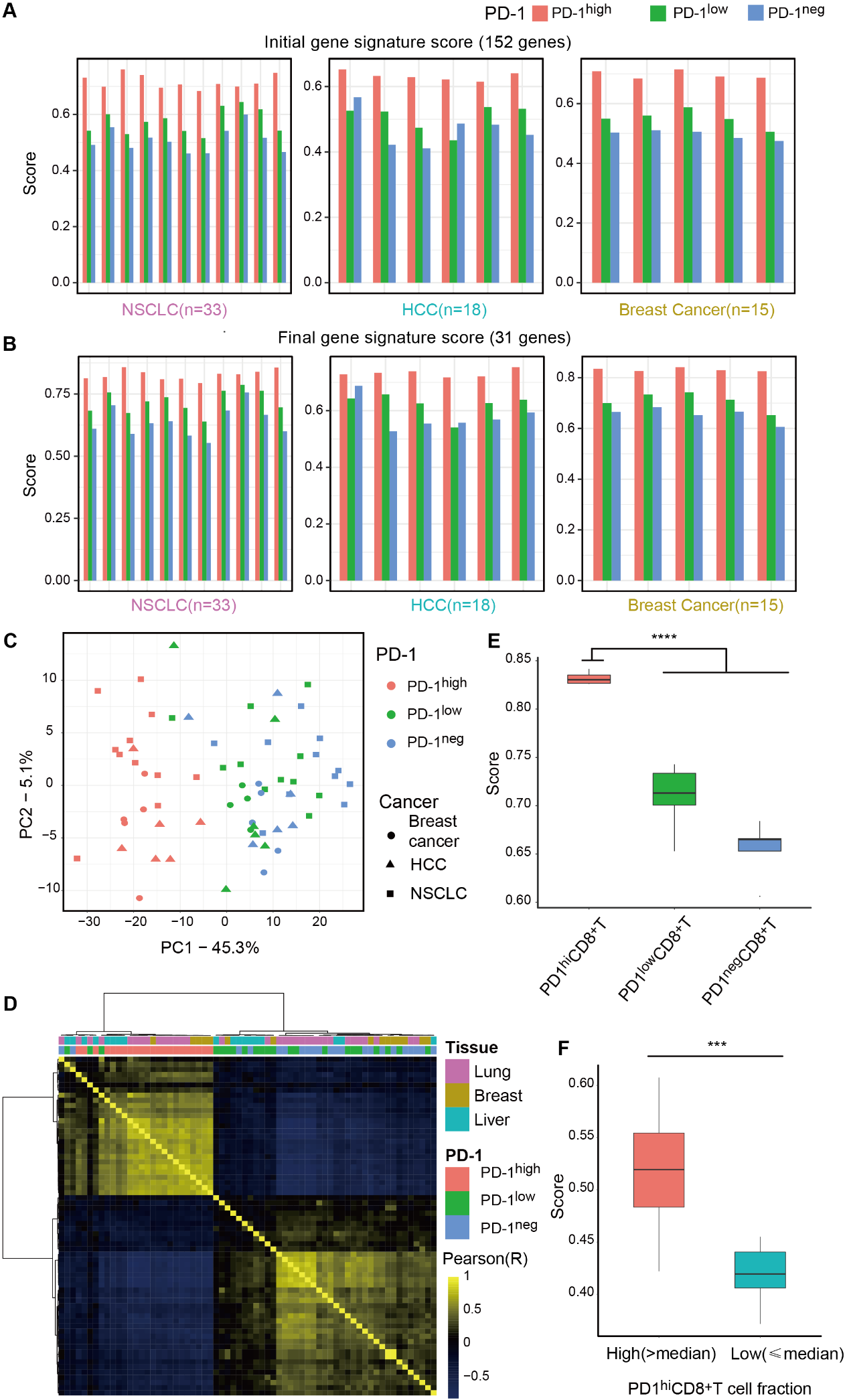


**Supplementary Figure 3**. Discrimination ability of our signature and the population similarity among non-small cell lung cancer (NSCLC, SRP108393), hepatocellular carcinoma (HCC, GSE111389) and breast cancer (SRP189910). (A, B). Both initial signature score (152 genes) and final signature score (31 genes) could accurately identify PD1^hi^CD8^+^T cells, reaching AUC=1 across three cancer types. (C). PD1^hi^CD8^+^T cells were clustered in principal component analysis. (D). Pearson correlation tests showed PD-1^hi^CD8^+^ T cells had similar transcriptional features and were tissue-agnostic among lung, liver and breast tissues. (E). In breast cancer, the PD1^hi^CD8^+^T cells were of highest signature scores. (****: Wilcoxon rank-sum test, *p*<0.0001). (F). In HCC bulk tumor tissue, patients with high fraction (> median) of PD1^hi^CD8^+^T cells had higher score than other patients (***: Wilcoxon rank-sum test, *p*=0.0009).


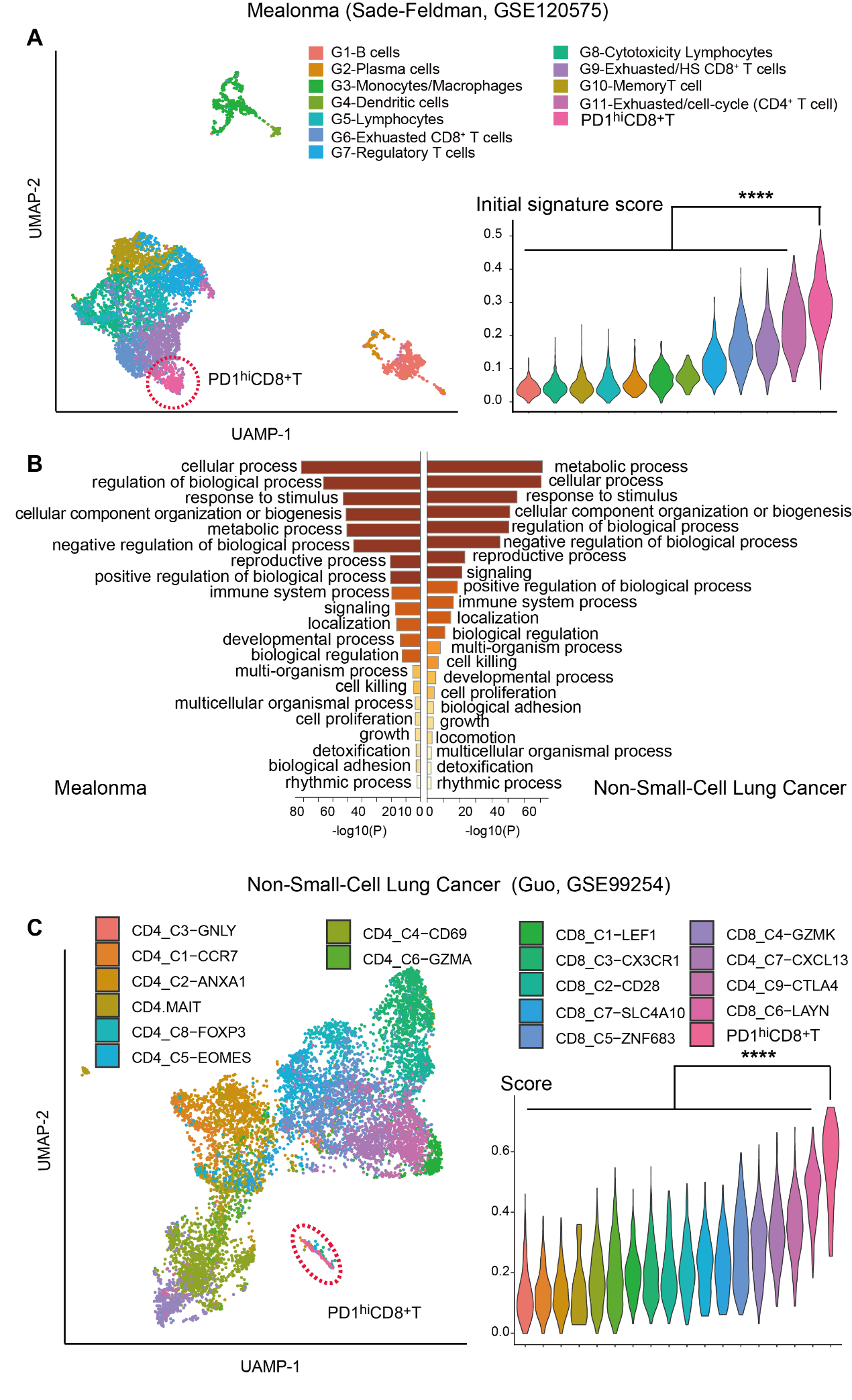


**Supplementary Figure 4**. Identification of PD1^hi^CD8^+^T cells in single-cell RNA-Seq datasets. (A). UMAP (Uniform manifold approximation and projection) plot of immune cells (Melanoma dataset from Sade-Feldman et al, GSE120575). PD1^hi^CD8^+^T cells were identified from the G11-exhausted/cell cycle cluster with highest initial signature score (calculated from the 152-genes initial signature), consistent with Figure 2E (****: *p*<0.0001). (B). Gene oncology (GO) enrichment analysis of high expression marker genes of PD1^hi^CD8^+^T cells. The marker genes were significantly enriched in cellular process, metabolic process and cell proliferation/growth among two studies. (C). UMAP plot of 18 T cell subsets (Non-small-cell lung cancer dataset from Guo et al, GSE99254). PD1^hi^CD8^+^T cells were identified from the CD8-C6-LAYN cluster with highest signature score (****: *p*<0.0001).


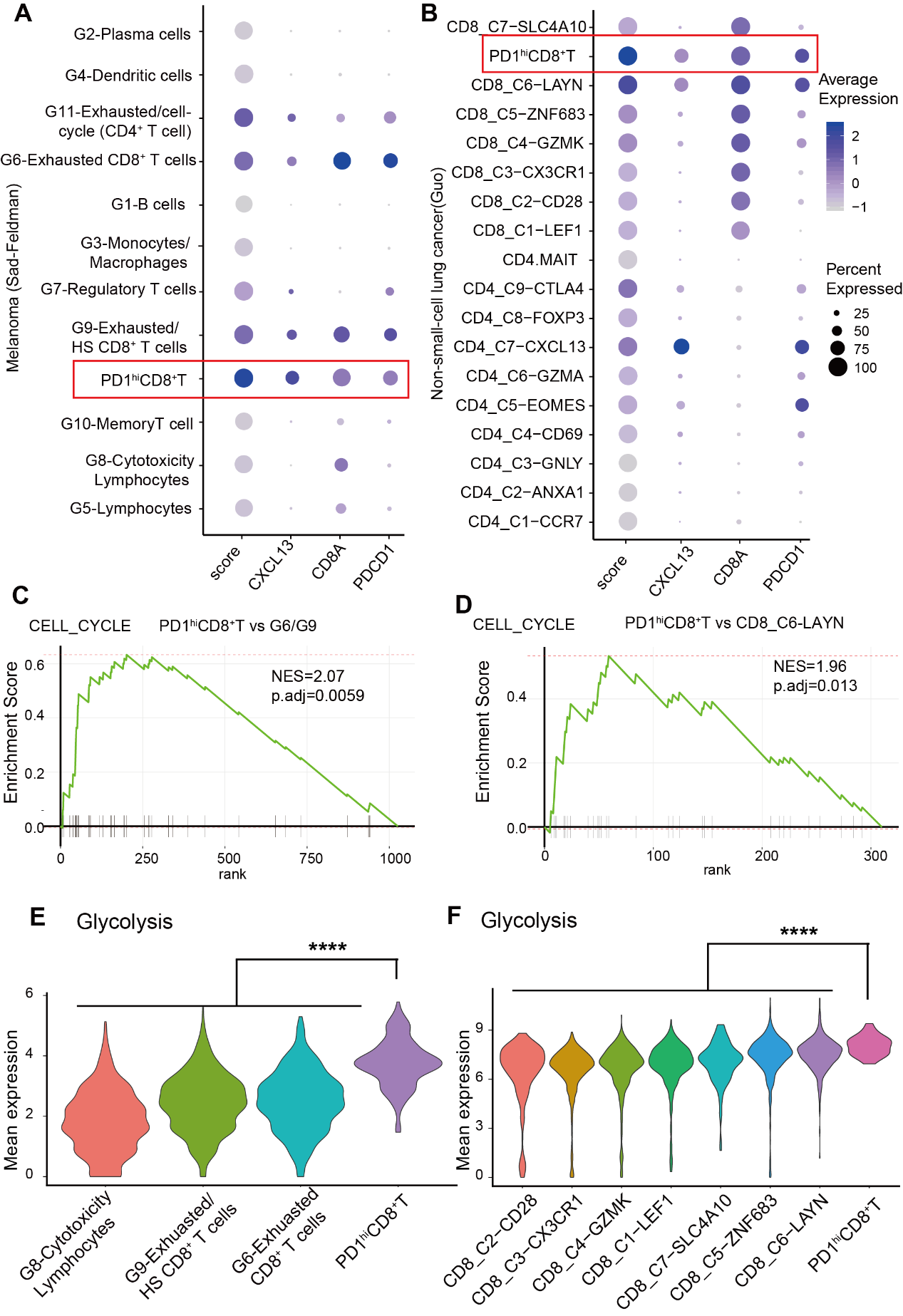


**Supplementary Figure 5**. Signature scores in different subsets and enrichment analysis. (A, B). Dot plot of average expression value of signature score, CXCL13, CD8A, PD-1 in two single-cell RNA-seq datasets. CXCL13 was a high expressed in PD1^hi^CD8^+^T cells (Melanoma: logFC=1.22, *p*.adj<0.0001; NSCLC: logFC=0.85, *p*.adj<0.0001, *p*.adj denotes p values adjusted by bonferroni correction in Seurat). (C, D). Gene set variation analysis (GSVA) of differentially expressed genes between PD1^hi^CD8^+^T cells and other CD8^+^T cells subsets with PD-1 expression but low signature score. (C). The cell cycle pathway was upregulated (Normalized enrichment score, NES=2.07, *p*.adj=0.0059, *p*.adj denotes p values adjusted by BH correction in GSVA) in PD1^hi^CD8^+^T compared to two exhausted CD8^+^T cells subsets (G6 and G9). (D). The cell cycle pathway was upregulated (NES=1.96, *p*.adj=0.013) in PD1^hi^CD8^+^T compared to exhausted CD8^+^T cells subset (CD8_C6-LAYN). (E, F). PD1^hi^CD8^+^T cells had increased glycolysis than other CD8^+^T cells (Wilcoxon rank-sum test; Melanoma: *p*<0.0001; NSCLC: *p*<0.0001).


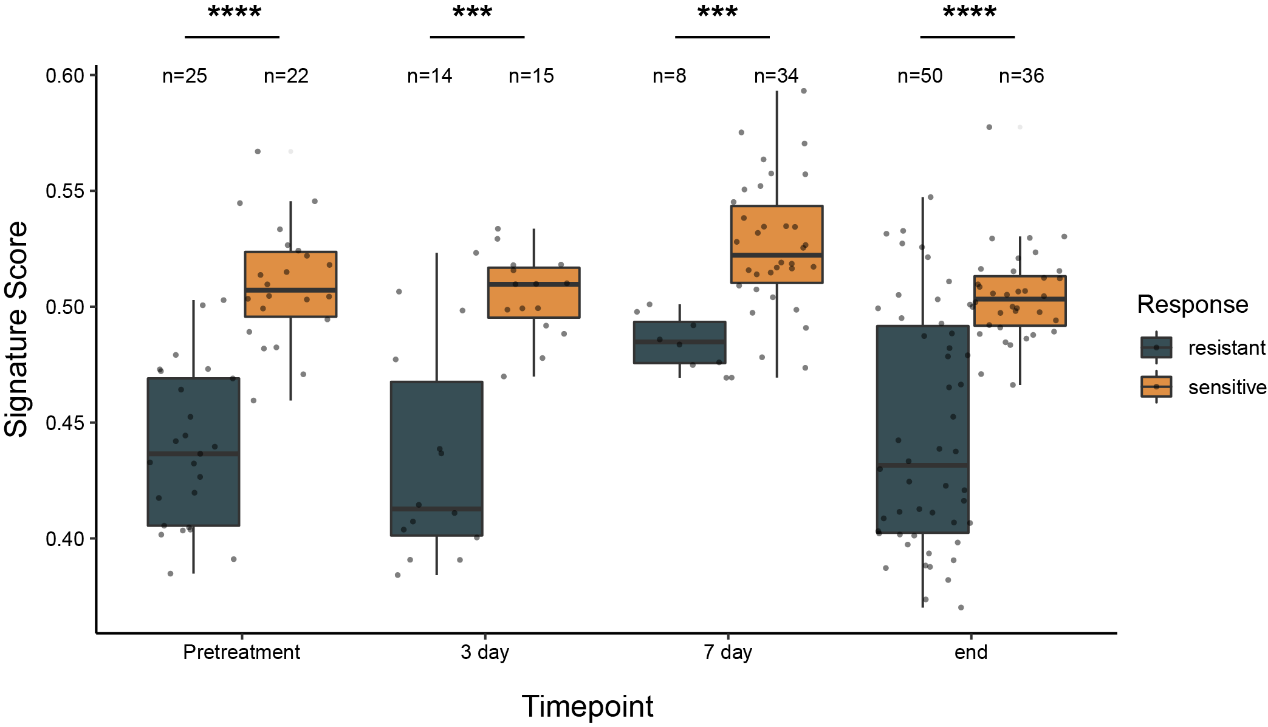


**Supplementary Figure 6.** The signature scores between resistant and sensitive mouse models of breast cancer treated by immune checkpoint inhibitors (GSE124821). Samples were collected at 4 timepoint, including pretreatment, 3 days after treatment, 7 days after treatment, end of treatment. Mouse genes were converted to human homologue by ‘biomaRt’ package.

**
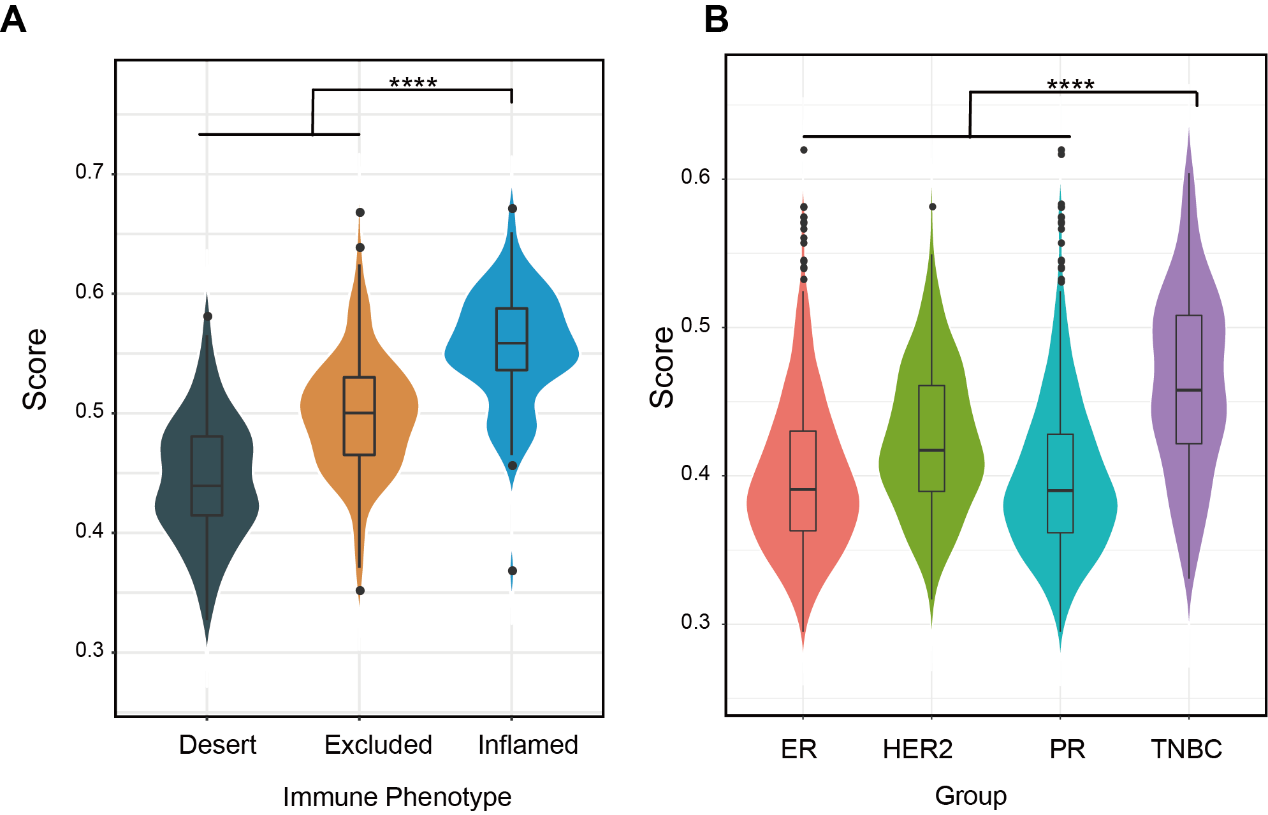
** **Supplementary Figure 7**. Signature score in different tumor subtypes and immune phenotypes. (A). Immune-inflamed tumors were of significant high signature scores. In the Mariathasan cohort, the immune phenotypes of cancer were divided into immune-inflamed, immune-excluded and immune-desert. (B). In breast cancer, the triple-negative breast cancer (TNBC) had higher score than human epidermal growth factor receptor-2 positive (HER2), hormone receptor positive (ER) and progesterone receptor positive (PR) subtypes. Wilcoxon rank-sum test, ****: *p*<0.0001.


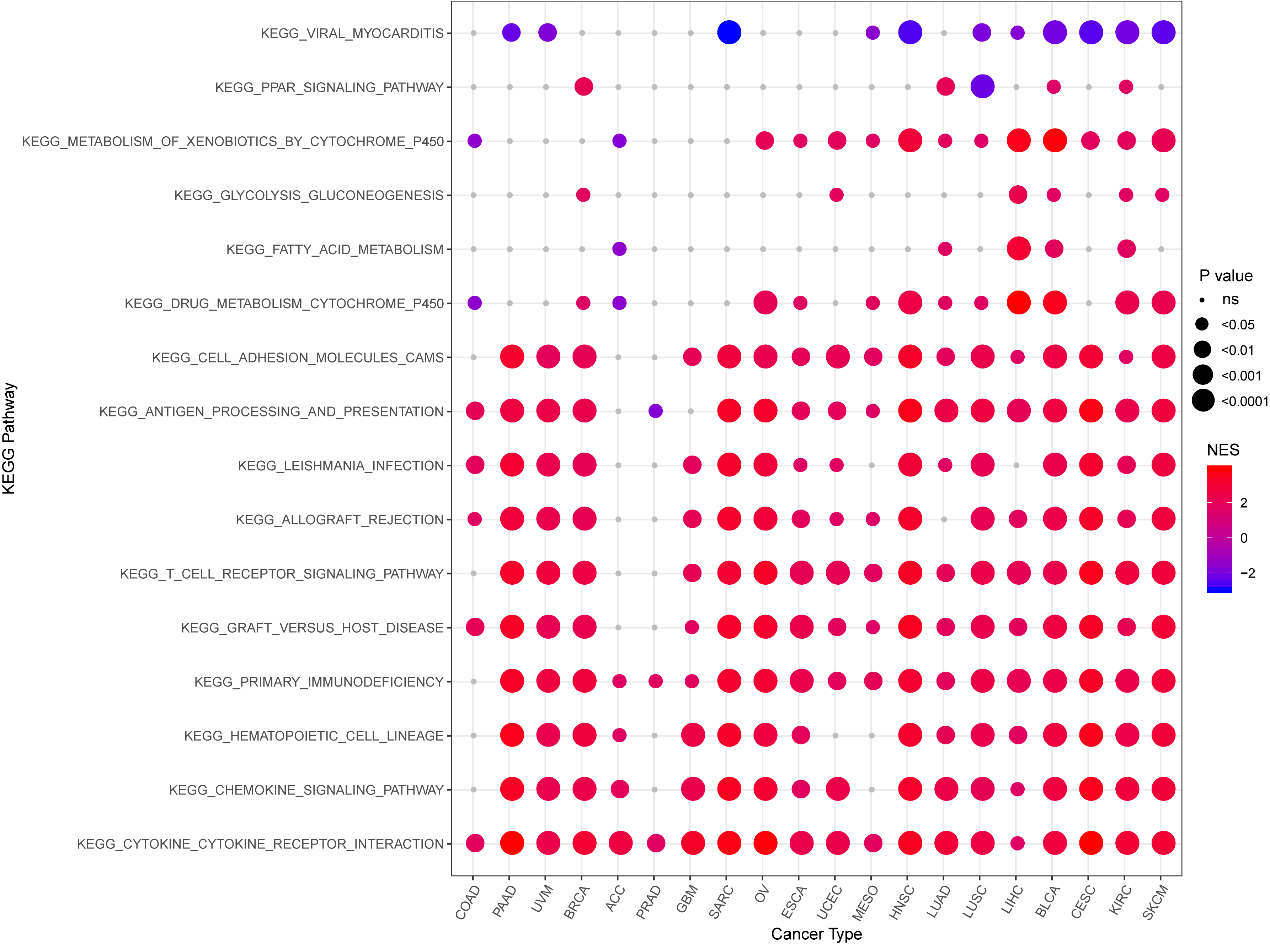


**Supplementary Figure 8**: KEGG pathway enrichment analyses of differentially expressed genes between high versus low signature score samples across cancer types. NES: normalized enrichment score; ns: none significance.


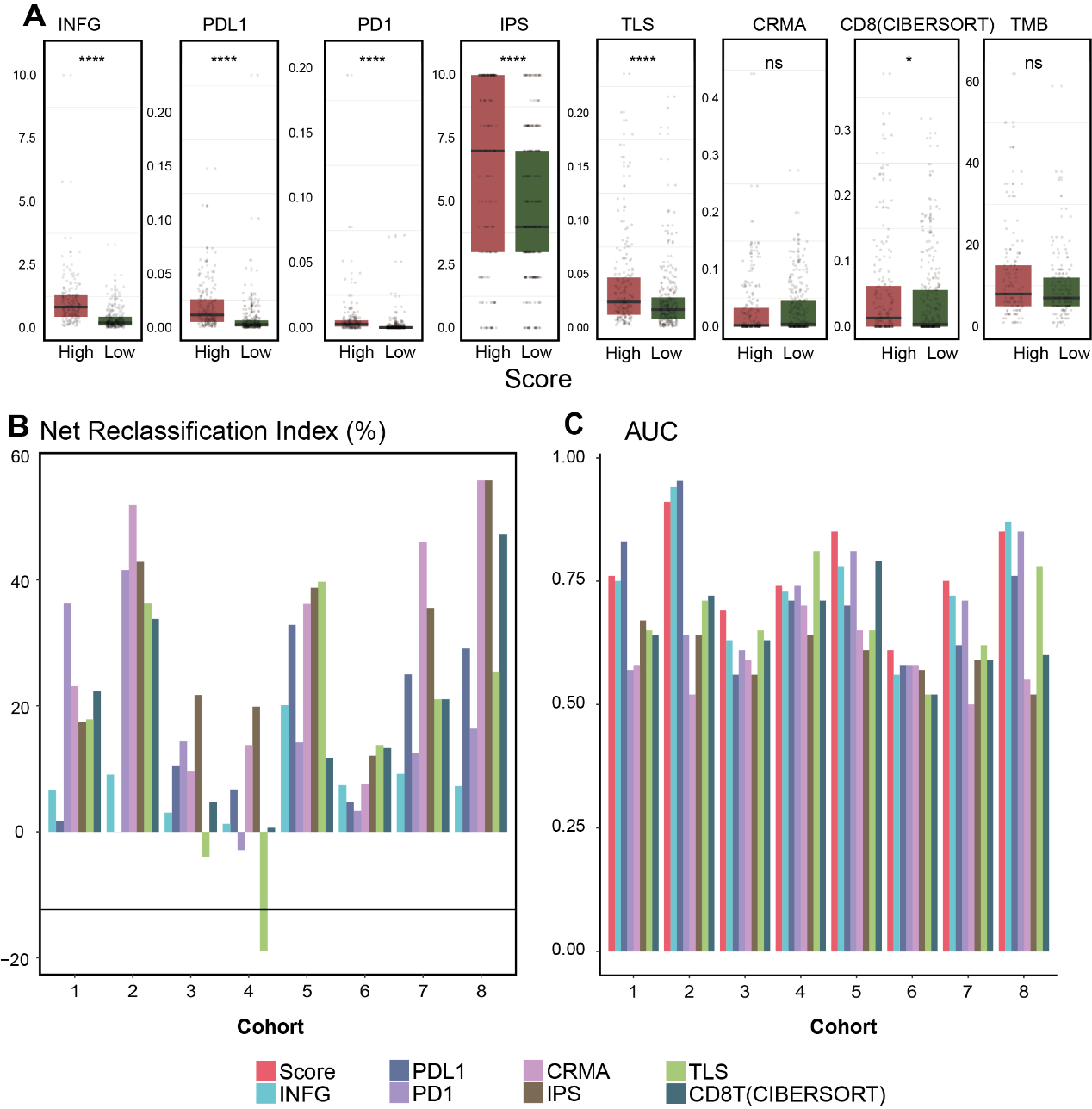


**Supplementary Figure 9**. Comparison with seven transcriptome immune checkpoint inhibitors (ICI) therapy biomarkers, including IFN-γ, PD-L1, PD-1, CRMA, TLS, IPS and CD8^+^ T CIBERSROT. (A) The associations between score and other biomarkers. ns: no significance; *: *p*<0.05; **: *p*<0.01; ***: *p*<0.001; ****: *p*<0.0001. (B) The net reclassification index of seven biomarkers compared to signature score. (C) The AUCs in eight cohorts. The eight cohorts were numbered in Supplementary Table 2.


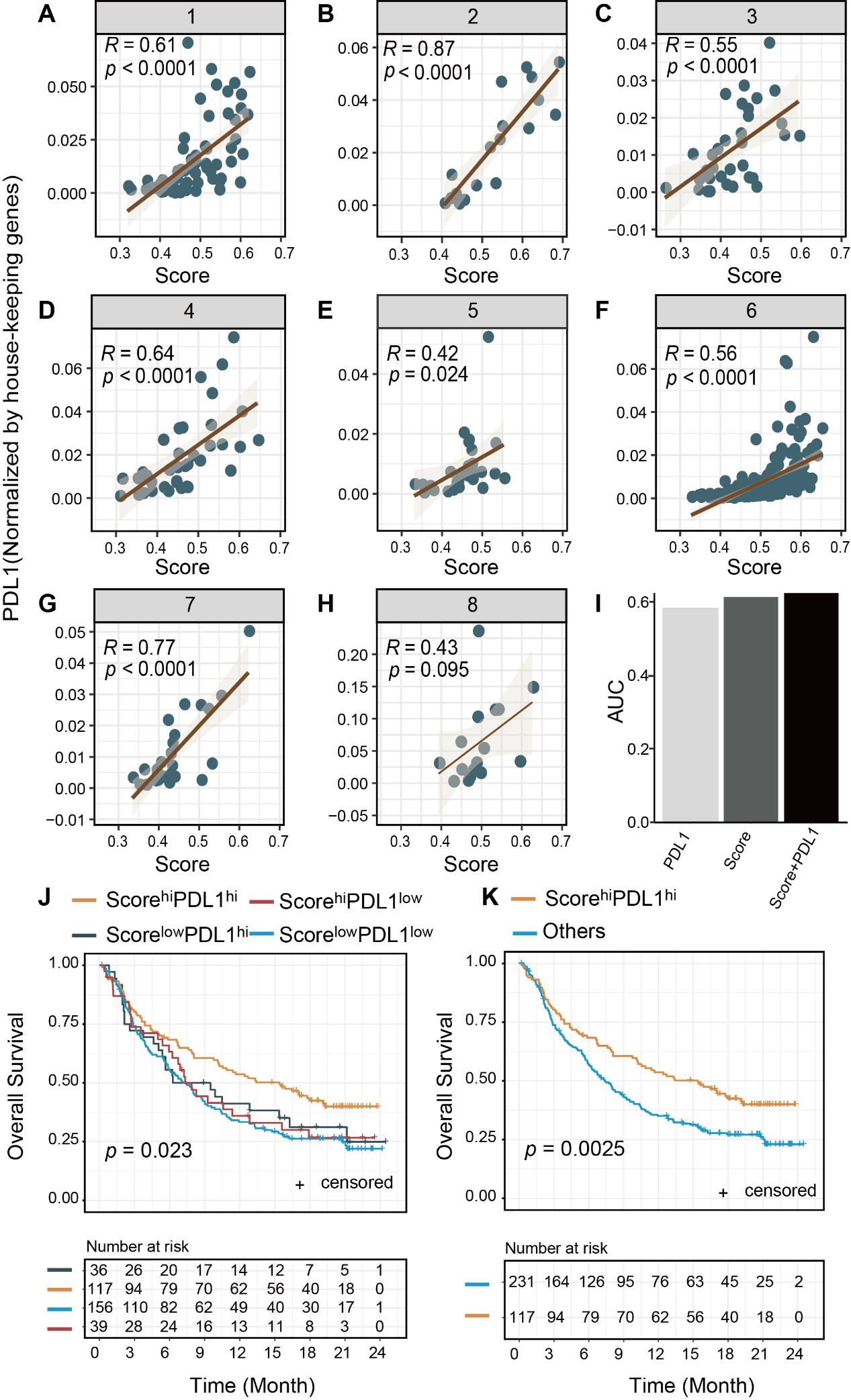


**Supplementary Figure 10.** The associations between PD-L1 and our score. (A-H). PD-L1 mRNA expression was correlated with our signature score in eight cohorts. (I). In the Mariathasan cohort, combining PD-L1 with our signature score could slightly increase the prediction value (PD-L1, AUC=0.58, Score, AUC=0.61, PD-L1+Score, AUC=0.63). (J) Significant differences exist between the 4 groups (PD-L1^high^Score^high^, PD-L1^low^Score^high^ , PD-L1^high^Score^low^ , PD-L1^low^Score^low^, *p*=0.023). (K). Patients in the PD-L1^high^Score^high^ group had better overall survival (E. Mariathasan dataset, *p=*0.0025) than other three groups.
